# Identification of novel point mutations in splicing sites integrating whole-exome and RNA-seq data in myeloproliferative diseases

**DOI:** 10.1002/mgg3.23

**Published:** 2013-07-07

**Authors:** Roberta Spinelli, Alessandra Pirola, Sara Redaelli, Nitesh Sharma, Hima Raman, Simona Valletta, Vera Magistroni, Rocco Piazza, Carlo Gambacorti-Passerini

**Affiliations:** 1 Department of Health Sciences, University of Milano–BicoccaMonza, Italy; 2 Hematology and Clinical Research Unit, San Gerardo HospitalMonza, Italy

**Keywords:** ABCC3, aCML, CML, DNAH9, GNAQ, HOOK1, KLHDC1, RNA-Seq, SMAD9, somatic mutations, splicing mutation, whole-exome sequencing

## Abstract

Point mutations in intronic regions near mRNA splice junctions can affect the splicing process. To identify novel splicing variants from exome sequencing data, we developed a bioinformatics splice-site prediction procedure to analyze next-generation sequencing (NGS) data (SpliceFinder). SpliceFinder integrates two functional annotation tools for NGS, ANNOVAR and MutationTaster and two canonical splice site prediction programs for single mutation analysis, SSPNN and NetGene2. By SpliceFinder, we identified somatic mutations affecting RNA splicing in a colon cancer sample, in eight atypical chronic myeloid leukemia (aCML), and eight CML patients. A novel homozygous splicing mutation was found in *APC* (NM_000038.4:c.1312+5G>A) and six heterozygous in *GNAQ* (NM_002072.2:c.735+1C>T), *ABCC3* (NM_003786.3:c.1783-1G>A), *KLHDC1* (NM_172193.1:c.568-2A>G), *HOOK1* (NM_015888.4:c.1662-1G>A), *SMAD9* (NM_001127217.2:c.1004-1C>T), and *DNAH9* (NM_001372.3:c.10242+5G>A). Integrating whole-exome and RNA sequencing in aCML and CML, we assessed the phenotypic effect of mutations on mRNA splicing for *GNAQ*, *ABCC3*, *HOOK1*. In *ABCC3* and *HOOK1*, RNA-Seq showed the presence of aberrant transcripts with activation of a cryptic splice site or intron retention, validated by the reverse transcription-polymerase chain reaction (RT-PCR) in the case of *HOOK1*. In *GNAQ*, RNA-Seq showed 22% of wild-type transcript and 78% of mRNA skipping exon 5, resulting in a 4–6 frameshift fusion confirmed by RT-PCR. The pipeline can be useful to identify intronic variants affecting RNA sequence by complementing conventional exome analysis.

## Introduction

It has been estimated that one third of the hereditary genetic diseases as well as many forms of cancer are caused by mutations resulting in the generation of altered transcript (Krawczak et al. 1992; Skotheim and Nees 2007; Fackenthal and Godley 2008; Gutierrez-Enriquez et al. 2009; He et al. 2009). Point mutations in intronic regions near mRNA splice junctions can affect mRNA splicing, altering the resulting RNA sequence and can have a profound effect on protein expression (Asselta et al. 2003; Chen et al. 2006; Wang and Cooper 2007).

The presence of well characterized donor and acceptor sites for RNA splicing at intron–exon borders makes these regions interesting targets for mutational screening analyses. Point mutations occurring in these regions typically lead to intron missplicing causing exon skipping or activation of cryptic splice sites. In cancer samples the molecular characterization of in-frame or out-of-frame splicing variants can potentially assist in the dissection of the oncogenic pathways.

In whole-exome sequencing techniques, the coverage of the intron–exon borders is typically high, usually comparable to that in exonic regions. There are many available tools that predict the functional effects of coding variants (Ramensky et al. 2002; Chun and Fay 2009; Kumar et al. 2009; Adzhubei et al. 2010; Liu et al. 2011) at whole exome-sequencing level and many more that analyze intronic splicing at single query level (Houdayer et al. 2008). However, an automated detection and functional prediction procedure of splicing variants from high-throughput sequencing data (HTS) is lacking. In order to identify novel and in-frame or out-of-frame splicing variants, we implemented a bioinformatics splice site prediction procedure to analyze next-generation sequencing data (SpliceFinder).

SpliceFinder (https://sites.google.com/site/splicefinder/) approach is based on two main steps. In the first step we annotate the variations detected by whole-exome sequencing and we predict from the nonexonic variants those with a damaging effect on RNA splicing in cancer samples and in the second step we confirm the splicing variants prediction by using two canonical splice-site analysis tools developed for single mutation analysis. Only the predictions found by three programs were accepted as putative splicing variants and sequenced by Sanger method. We assumed that three similar outcomes are able to predict the damaging effect on RNA splicing as previously proposed in the decision tree for single query analysis (Vreeswijk et al. 2009).

In addition, to assess the phenotypic effects of the somatic mutations on mRNA splicing and gene expression level, we combined the DNA mutational screening analysis with RNA-Seq profiles on leukemic cells. The RNA splicing maps were obtained by using TopHat (Trapnell et al. 2009), a splice junction mapper algorithm, and the whole-gene expression profile analysis was performed by SAMMate (Xu et al. 2011). When possible we confirmed the novel mRNA splicing by reverse transcription-polymerase chain reaction (RT-PCR).

To test the ability of SpliceFinder to identify splicing variants, we initially analyzed the whole-exome sequencing data from a colon cancer sample matched to peripheral blood (PB) as control sample that surprisingly did not show coding variants in genes associated with colon cancer such as *APC* and *β-catenin*. By using our procedure we identified a novel somatic, intronic mutation affecting a splicing site in *APC*. Then we applied the SpliceFinder to whole-exome sequencing data from eight chronic myeloid leukemia (CML) and eight atypical chronic myeloid leukemia (aCML) (Vardiman et al. 2009) patient samples including matched autologous normal lymphocytes; RNA-Seq data were used to evaluate the relative mRNA abundance (SRA061202, GSE42146) and the splicing gene profiles.

We conclude that this procedure allows the identification of novel splicing mutations in cancer genomes and we show the applicability of SpliceFinder as a tool to complement exome analysis to identify gene splicing abnormalities.

## Materials and Methods

### Patients and samples

A formalin fixed paraffin embedded (FFPE) colon cancer sample with a percentage of tumoral cells greater than 80% (evaluated at the microscope by the anatomical pathologist) was compared with the PB of the same patient used as negative control.

For the eight CML and eight aCML cases, bone marrow or peripheral blood were collected at diagnosis after informed consent, before any therapy. CML patients showed the BCR-ABL fusion gene and aCML patients showed normal cytogenetic analysis. The diagnosis of aCML were performed according to the WHO classification (Vardiman et al. 2009).

Myeloid cells were evaluated by fluorescence-activated cell sorting (FACS) analysis and constituted more than 80% of total cells. Lymphocytes, obtained from PB samples of patients in remission or culturing cells with 2,5 μg/mL Phytohemagglutinin-M (PHA-M) (Roche Diagnostics GmbH, Germany) and 200 UI/mL Interleukin-2 (IL-2) (Aldesleukin, Novartis – Switzerland) for 3–4 days followed by 2–3 weeks incubation with only IL-2, were used as normal cells. The phenotype was evaluated by FACS analysis and lymphoid cells resulted to be more than 80% of the total.

### Exome sequencing

Genomic DNA from the FFPE tissue block was extracted with RecoverAllTM Total Nucleic Acid Isolation kit (Ambion, 2130 Woodward Street, Austin, TX 78744) using 5 × 5 μm unstained sections. Genomic DNA from leukemic cells and normal lymphocytes was extracted with PureLinkTM Genomic DNA Kit (Invitrogen, Life technology, Grand Island, NY). The exome libraries were generated starting from 1 μg of gDNA (2 μg for the FFPE sample) and using Illumina TruSeqTM Exome Enrichment Kit (FC-121-1008; Illumina, San Diego, CA) with fragment size of 200–300 bp. The libraries were sequenced using the Illumina Genome Analyzer IIx, with 76 bp paired-end reads and the Illumina TruSeqTM SBS kit v5 (FC-104-5001).

### RNA sequencing

Total RNA was extracted from leukemic cells with TRIzol® Reagent (Invitrogen, Life technology, Grand Island, NY). The libraries were prepared using Illumina Tru-SeqTM RNA Sample Preparation Kit (FC-122-1001) protocol starting from 2 μg of total RNA with fragment size of 400–500 bp. The libraries were subsequently sequenced using an Illumina Genome Analyzer IIx with 76 bp paired-end reads and the Illumina TruSeqTM SBS kit v5 (FC-104-5001).

### Whole-exome sequencing analysis

Image analysis and base calling were performed using the Illumina Real Time Analysis Software RTA v1.9.35. The binary bcl files were converted to qseq by using the Off-Line Basecaller OLB v1.9.0. Qseq files were deindexed and converted in the Sanger-FastQ file format using in-house scripts. FastQ sequences were aligned to the human genome database (NCBI36/hg18) using the Burrows–Wheeler-based BWA alignment tool (Li and Durbin 2009) within the Galaxy framework (Giardine et al. 2005; Blankenberg et al. 2010; Goecks et al. 2010). The alignment files in the SAM format were analyzed by SAMtools (http://samtools.sourceforge.net/, [Li et al. 2009]). Uniquely mapped reads, with a mapping quality major than 30 and mapped in proper pair were accepted for the downstream analysis. Duplicated paired-ends reads were excluded from the analysis, the results were then converted in the Pileup format.

### Pipeline for somatic mutations discovery

Pileup data generated from paired cancer and control samples were cross-matched to identify the candidate somatic mutations, as variations occurring only in the cancer genome but not in paired control sample, by using in-house software in C# language (Piazza et al. 2013). To obtain robustness of mutation detection we filtered variations in cancer pileup file with read coverage ≥20, frequency of substitution ≥6, percentage of substitution ≥25%, Phred (Ewing and Green 1998) read quality score ≥30, corresponding to a probability of incorrect base ≤0.001. Finally, variations present in matched healthy pileup file with a frequency lower or equal than 10% were tolerated.

### Splice-site prediction analysis

SpliceFinder is a method for rapid functional prediction of splicing variants starting from a large set of somatic mutations obtained by whole-exome sequencing analysis. The SpliceFinder methodology is a bioinformatics integrated procedure based on two public functional annotation tools for HTS analysis, ANNOVAR (Wang et al. 2010) and MutationTaster (Schwarz et al. 2010) and two canonical splice-site prediction software programs for single splicing analysis, SSPNN (http://www.fruitfly.org/seq_tools/splice.html, Reese et al. 1997) and NetGene2 (http://www.cbs.dtu.dk/services/NetGene2/, Brunak et al. 1991; Hebsgaard et al. 1996). In order to obtain the noncoding mutations near exon–intron border we used ANNOVAR software (vs 2013 Feb11) and to predict the splicing variants that affected physiological splicing we used MutationTaster software based on statistical Naive Bayes classifier. We then confirmed the results by querying SSPNN and NetGene2 using default parameters. Only the predictions found in all three programs were accepted as putative splicing variants and sequenced by Sanger method.

Because we are interested in splicing variants analysis, from all coding and noncoding annotated variants we collected only the splicing variants within 20-bp of a splicing junction, in conserved regions, not previously reported in dbSNP or in 1000Genome Project and not in segmental duplication regions. By using ANNOVAR we were able to annotate the mutations at gene level; identify whether the variant hits exons, introns, or splicing within 20 bp away from an exon-intron boundary (default window size equal to 2) or hits intergenic regions or noncoding RNA genes; identify variants that are reported in dbSNP130, or are common SNPs (MAF >1%) in the 1000 Genome Project (pilot data 2010 July release) and discover variants in the most conserved genomic regions among 44 vertebrate species or in segmental duplication regions (likely to be affected by genotype calling issue). The 44 species conservation track (phastConsElements44way) was used as a measure of evolutionary conservation among 44 vertebrate species.

Then, to predict the splicing mutations that affected donor and acceptor splice sites and to evaluate the efficiencies of physiological splicing sites in mutant genomic sequence we used MutationTaster that is able to predict the disease-causing potential on both exonic and nonexonic variants. This is a next-generation sequencing tool for a rapid evaluation of thousands of DNA sequence alterations and NGS data and it has the advantage to analyze both exonic and nonexonic variants such as splice sites, poly(A) signal, Kozak consensus sequences. In addition, MutationTaster evaluates the disease-causing potential of DNA sequence alterations based on statistical naive Bayes classifier trained and validated on known models of disease mutations and polymorphisms effects (Hand and Yu 2001). MutationTaster calculates the probability of an alteration to be either a disease causing mutation or a neutral variant. A probability value of prediction close to 1 means a high-quality prediction. In order to pickup the most probability splicing variants, we analyzed the sequence alterations identified in the previous step by the batch query analysis, which is also able to report the classification prediction of disease causing or polymorphism, the probability of prediction, the dbSNP annotation, HapMap genotype frequency, evolutionary conservation score, and the protein features that can be affected. To run the batch query analysis, we converted the physical genomic position from the NCBI36/hg18 to the GRCh37/hg19 build by the Lift Genome Annotations tool in Human Genome Browser (UCSC, http://genome.ucsc.edu) and then we ran a batch query analysis by QueryEngine system (http://www.mutationtaster.org/StartQueryEngine.html). In our analysis we accepted as a true positive or good candidate the putative splicing variants with a probability of disease causing prediction greater than 0.9 not yet annotated in dbSNP and preferably in a conserved evolutionary region.

In the next steps, the splicing predictions accomplished by MutationTaster were also confirmed by two other splice-site analysis tools that are currently used to predict the presence and efficiencies of splice donor and acceptor sites on single queries. We assumed that a similar outcome of three different prediction software would be sufficient to predict the damaging effect of splicing somatic variant on pre-mRNA splicing, as suggested in the decision tree for the single query analysis (Vreeswijk et al. 2009). Then, the efficiencies of constitutive donor and acceptor sites were evaluated in the wild type and mutant sequence by querying SSPNN and NetGene2 by using default parameters. The output was accepted if a constitutive splice site was recognized. Finally, all the putative splicing variants were checked for absence in dbSNP and in COSMIC (Forbes et al. 2010), and then confirmed by Sanger sequence.

### Transcriptome sequencing analysis

Image analysis and base calling were performed using the Illumina Real Time Analysis Software RTA v1.9.35. The binary bcl files were converted to qseq by Off-Line Basecaller OLB v1.9.0. Qseq files were deindexed and converted in the Sanger-FastQ file format using in-house scripts. FastQ sequences were aligned to the human genome database (NCBI36/hg18) by TopHat algorithm (Trapnell et al. 2009) (vs 1.2.0), a splice junction mapper for RNA-Seq data, that can map the reads across the junctions, by using default parameters. The reads were mapped according to the gene and splice junctions model provided in the Human Ensembl annotation GTF file (Homo_Sapiens.NCBI36.54.GTF) downloaded from Ensembl release 54 (http://ftp://ftp.ensembl.org/pub/release-54/gtf/homo_sapiens/). TopHat aligns the RNA-Seq reads through the genome using Bowtie (Langmead et al. 2009) and then maps the initially unmappable reads (IUM) to the known splice junctions sequences supplied by the annotation GTF file. A splice junctions map for whole transcriptome in CML and aCML patients was inferred by TopHat, which allowed to identify the exon junction map for wild-type and mutant sequence and visualized by the Integrated Genomic Viewer (IGV) (Robinson et al. 2011) or UCSC Genome Browser. The novelty of aberrant splicing mRNA were confirmed by manually inspection of the Transcription database in Ensembl release 71 – April 2013. The quantitative gene expression profiles were estimated by SAMMate (Xu et al. 2011) (vs 2.6.1) by using default parameters. SAMMate calculates the expression values for each gene taking into account the reads both mapped on exons or on exon–exon junctions. The expression values for paired-end data were measured in Fragments Per Kilobase of exon model per million mapped reads (FPKM) (Mortazavi et al. 2008) which is a normalized measure of exonic read density and a measure of concentration of a transcript. The human Ensembl gene annotation file vs 54 was used to infer the expression values. Starting from the Binary sequence Alignment Map file (accepted_hits.BAM) a matrix of FPKM expression values for 36,655 unique Ensembl Gene were obtained by SAMMate.

### Sanger sequencing

To validate the somatic point mutations identified by whole-exome sequencing, two primers, upstream and downstream the mutation, were designed using Vector NTI software, and used in a polymerase chain reaction (PCR) (FastStart High Fidelity PCR System, Roche Applied Science, Mannheim, Germany) to amplify a region of 200–500 bp. These amplicons were then sequenced by Sanger Sequencing and the presence of the mutation identified using Chromas 2 Software.

### RT-PCR

Total RNA was extracted from leukemic cells with TRIzol® Reagent (Invitrogen, Life technology, Grand Island, NY). One microgram of RNA was retrotranscribed using the MultiScribe™ Reverse Transcriptase (Invitrogen, Life technology, Grand Island, NY) according to manufactory protocol. The cDNA obtained for CML patient no. (pt.) 1 was subsequently used for PCR amplification of *GNAQ* (NM_002072) using the following primers: GNAQ_ex4_fwd (5’-TACTATCTTAATGACTTGGACCG-3’) and GNAQ_ex6_rev (5’-TCCATCATATTCTGGGAAGT-3’). Sanger sequencing of the amplicon was performed using GNAQ_ex4_fwd primer. The cDNA obtained for aCML pt. 6 was subsequently used for PCR amplification of *HOOK1* (NM_015888) using the following primers: HOOK1_intr17_fwd (5’–CAGTCTCCATGCTTTTTCTACC-3’) and HOOK1_ex19_rev (5’–GCTTCAAGTTCATTGATCTTTTGTA-3’). Sanger sequencing of the amplicon was performed using HOOK1_ex19_rev primer.

## Results

### Splicing variant study

Standard whole-exome sequencing analysis performed on a colon cancer specimen revealed the presence of 319 coding SNVs. Of them, 144 were annotated nonsynonymous and not reported in dbSNP by SIFT (Kumar et al. 2009). None of these variants occurred in genes associated with colon cancer, such as *APC* and *β-catenin*. Therefore, after applying our SpliceFinder procedure we were able to identify a previously unreported somatic G->A transition, affecting position +5 of the donor splice site in the intron between exon 10 and 11 of *APC* (c.1312+5G>A, OMIM# 611731, NM_000038, NCBI36.1 nomenclature), with a mutation rate of 81% (chr5:112,182,944-112,182,945;G/A) compatible with an homozygous status. The absolute read coverage in tumor and normal samples was, respectively, 90 and 63 and the mutation frequency was 73 and 0 (Fig. S1). The loss prediction of constitutive donor site on RNA splicing obtained by MutationTaster in the mutant sequence (score 1) was confirmed by SSPNN and NetGene2. Both tools recognized the canonical donor site in the wild-type sequence (SSPNN score 0.93 and NetGene2 score 0.864) and the loss of constitutional donor site in the mutant sequence affecting *APC* splicing.

Subsequently, we applied our SpliceFinder procedure to a CML dataset where we found a total of 8 (pt. 1), 17 (pt. 2), 1728 (pt. 3), 896 (pt. 4), 631 (pt. 5), 1203 (pt. 6), 382 (pt. 7), 16 (pt. 8) somatic variations in noncoding regions with minimum read depth equal to 20 and minimum percent of mutation equal to 25% (Table S1). Of them 1, 2, 96, 48, 64, 110, 32, 0 were localized within 20 bp from a splicing junction. Among these variants, the splicing prediction analysis suggested the presence of three splicing variants impacting the canonical AG/GT splice sites, identified, respectively, in pt. 1, pt. 4, and pt. 5 (Table 1). No evidence of splicing variants could be found by SpliceFinder in the other CML patients (pt. 2, pt. 3, pt. 6, pt. 7, pt. 8).

**Table 1:** Splicing prediction summary for CML and aCML samples

Gene	Patient ID	Locus	Mutation	Absolute coverage T (N)	Mutation frequency T (N)	Mutation fraction T(N) (%)	Splicing prediction
GNAQ	Ph+001	9,79599197,79599198,1,C/T	c.735+1C>T	23 (65)	8 (0)	35% (0%)	Donor lost
ABCC3	Ph+004	17,46100788,46100789,1,G/A	c.1783-1G>A	59 (36)	30 (0)	51% (1%)	Acceptor lost
KLHDC1	Ph+005	14,49265391,49265392,1,A/G	c.568-2A>G	93 (14)	48 (1)	52% (7%)	Acceptor lost
SMAD9	Ph-005	13,36325812,36325813,1,C/T	c.1004-1C>T	27 (16)	14 (0)	52% (0%)	Acceptor lost
HOOK1	Ph-006	1,60103421,60103422,1,G/A	c.1662-1G>A	186 (153)	87 (0)	47% (0%)	Acceptor lost
DNAH9	Ph-007	17,11715832,11715833,1,G/A	c.10242+5G>A	37 (43)	19 (0)	51% (0%)	Donor lost

Description of six splicing mutations according to NCBI 36.1 nomenclature. T (N), tumor and matched normal sample.

**Figure 1:**
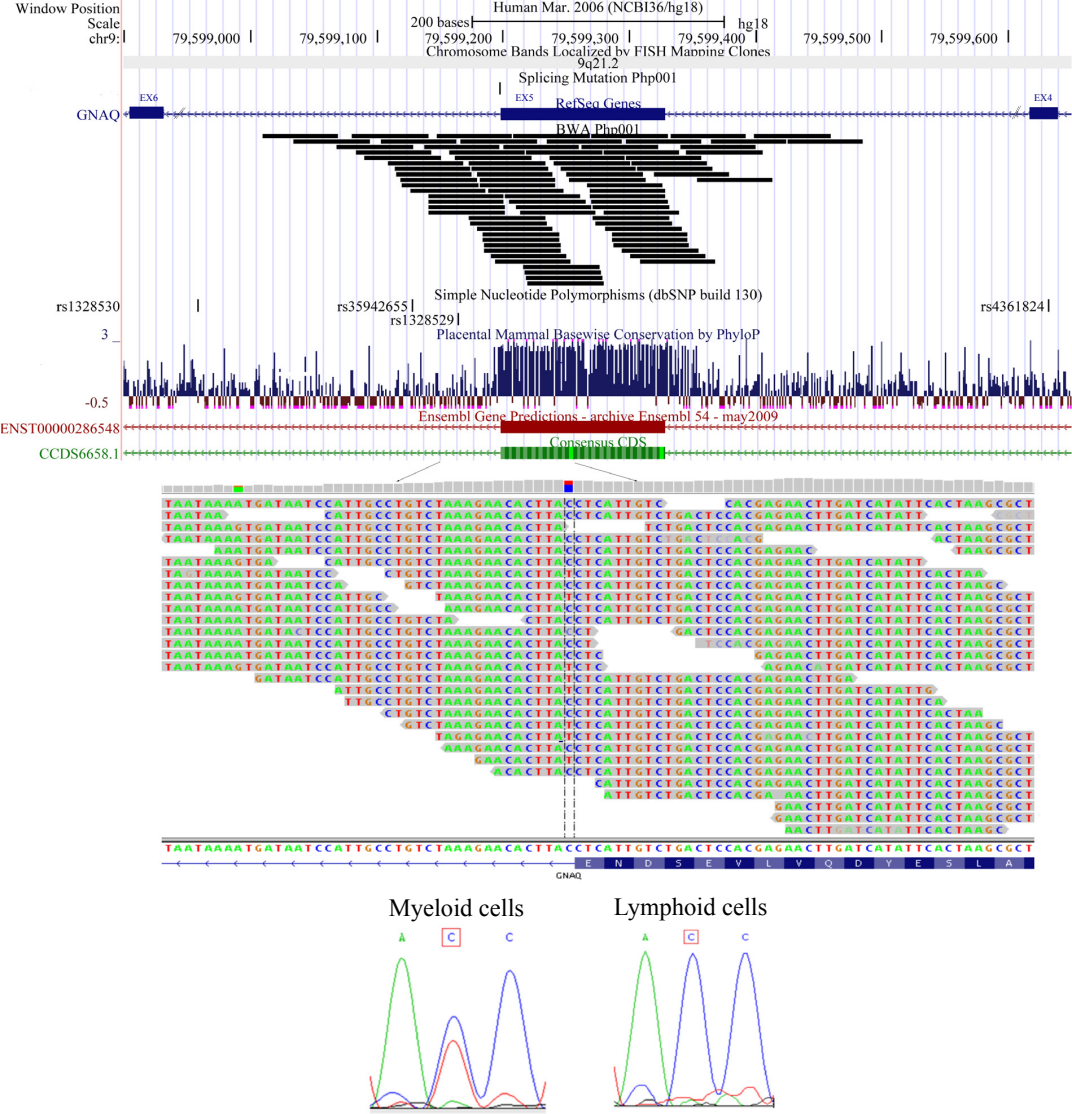
*GNAQ* (NM_002072.2:c.735+1C>T) splicing mutation near the 5’ donor splice site at position +1 in the intron between exons 5 and 6 in UCSC panel and somatic mutation frequency of 35% in IGV visualization. Below, the Sanger validation.

**Figure 2:**
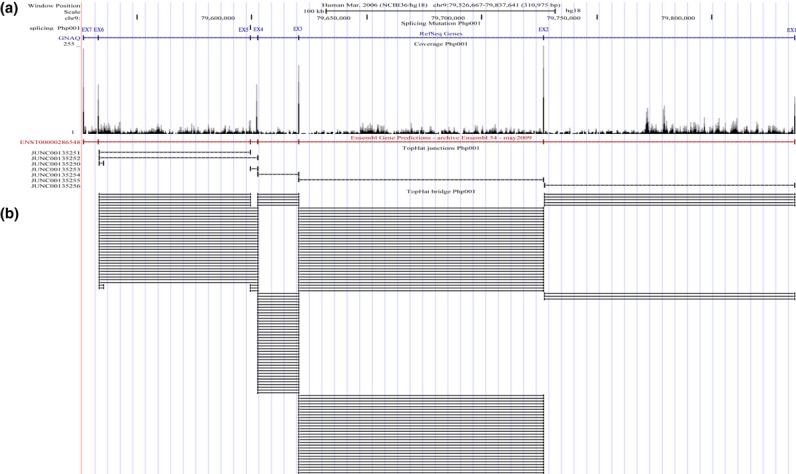
(a) RNA-Seq read coverage of *GNAQ* (NM_002072) in Ph+001. (b) RNA-seq showed 28 junction reads between exon 4 and exon 6 resulting in exon 4 to exon 6 frameshift fusion (78% mutant sequence), five junction reads between exons 5 and 6, and three junction reads between exons 4 and exons 5 (22% wild-type sequence).

**Figure 3:**
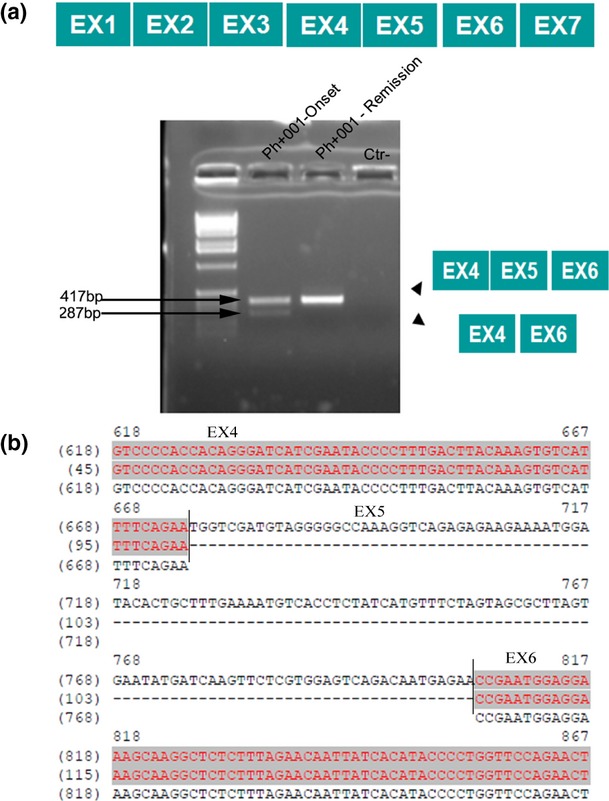
(a) PCR product of *GNAQ* (NM_002072) (from exon 4 to exon 6) from cDNA of tumor and matched remission Ph+001 sample showed the presence of different bands: the wild type one (417 bp) and the shorter aberrant one (287 bp) present only in the tumor sample. (b) Sanger sequencing of the aberrant *GNAQ* product showed complete loss of exon 5.

In pt. 1, SpliceFinder analysis predicted the loss of a donor splicing site near the 5’ donor, at position +1 in the intron between exon 5 and 6 of the *GNAQ* (OMIM# 600998) proto-oncogene (NM_002072.2:c.735+1C>T, NCBI36.1 nomenclature). The somatic variant was present with a frequency of 35%. The presence of this mutation was confirmed by Sanger sequencing (Fig. 1, Table S2). RNA-Seq analysis showed that 78% of GNAQ mRNA effectively skipped the upstream exon 5, resulting in a 4–6 frameshift fusion (Fig. 2, Fig. S2A, Table 2), which likely destroys the GTPase activity of GNAQ. RT-PCR of tumor mRNA showed the presence of two amplicons: of them, one was in common with the matched control; the other one, shorter than the wild type, was compatible with the length of the exon skipping RNA and wasn't present in the matched remission RNA sample. By sequencing the two tumor GNAQ transcripts, we found a wild type isoform and a new fusion transcript arising from exon 5 deletion resulted in a premature stop codon (Fig. 3, see M&M). No evidence of GNAQ exon 5 deleted RNA was found in CML patients who lacked the intronic mutation (Fig. S2B). *GNAQ* is ubiquitously expressed in all tissues and it shows high expression level (32.938 FPKM) in mutated sample and falls at the 90th percentile among the gene expression values from the CML data set. The expression level is not different in the patient with abnormal splicing compared with the others (mean expression value of 42.885 FPKM in *GNAQ*-unmutated CML samples). No skipping of exon 5 was found in additional aCML patients.

**Table 2:** Point mutations within splice sites and their effect on mRNA splicing scored by MutationTaster, SSPNN, and NetGene2 (NCBI36.1 nomenclature)

Gene	Patient ID	Mutation	MutationTaster	SSPNN w.type/mutant	NetGene2 w.type/mutant	Exon length (bp)	Reads counts (nr)	RNA-seq expression level (FPKM)	Comment
GNAQ	Ph+001	c.735+1C>T	1	1.00/–	0.997/–	2160	2843	32.938	22% wt RNA, 78% skipping ex5^1^, out of frame
ABCC3	Ph+004	c.1783-1G>A	1	0.91/–	0.390/–	5155	859	6.109	58% wt RNA, 37% intron retention, 5% activation cryptic site, out of frame
KLHDC1	Ph+005	c.568-2A>G	1	0.93/–	0.946/–	2644	72	0.939	Low expression
SMAD9	Ph-005	c.1004-1C>T	1	0.98/–	0.988/–	5558	42	0.379	Low expression
HOOK1	Ph-006	c.1662-1G>A	1	0.94/–	0.877/–	5861	344	3.767	58% wt RNA, 42% intron retention,^1^ mutation detected by RNA-Seq, out of frame
DNAH9	Ph-007	c.10242+5G>A	0.999	0.95/–	0.949/–	14087	0	0	Not expression

Transcript quantification by RNA-seq analysis. –, the constitutive splice site is not recognized in mutant sequence.

1 Data confirmed by RT-PCR.

In pt. 4, we identified a somatic mutation near the 3’ acceptor splice site at position −1 in the intron between exons 13 and 14 of *ABCC3* (OMIM#604323) gene (NM_003786.3:c.1783-1G>A); it was present with a frequency of 51%. The somatic mutation was confirmed by Sanger sequencing (Fig. S3A, Table S2). In this case, SpliceFinder analysis predicted the loss of a physiologic acceptor site causing exon skipping or an activation of a new cryptic site. The RNA-Seq analysis showed that 58% of *ABCC3* mRNA was wild type, 37% retained intron 13%, and 5% was characterized by the presence of a new cryptic splice site five bases within the exon 14 (Fig. S3B, Table 2). No evidence of this event was found in CML and aCML patients not mutated in *ABCC3* (Fig. S3C). However, RNA-Seq did not show the splicing mutation in retained intron sequence of pt. 4. The *ABCC3* expression value was equal to 6.109 FPKM (58th percentile in pt. 4) similarly to the mean expression value of *ABCC3*-unmutated CML samples (mean expression value 5.403 FPKM, 62nd percentile).

In pt. 5, we found a splicing mutation in the *KLHDC1* (OMIM#611281) gene (NM_172193.1:c.568-2A>G), near the 3’ acceptor splice site at position −2 in the intron between exons 6 and 7 with a frequency of 52%. The Sanger sequencing confirmed the somatic mutation (Fig. S4A, Table S2). SpliceFinder predicted a loss of acceptor splice site (Table 2) but the low expression level of the *KLHDC1* gene (0.939 FPKM) in the leukemic sample prevented us from generating a reliable exon junction map.

In the aCML data set we found 7, 135, 3, 74, 59, 128, 27, 45 somatic variations in noncoding regions, respectively, from pt. 1 to pt. 8. Of them 2, 9, 0, 1, 1, 4, 1, 2 were localized within 20 bp from a splicing junction (Table S1) and only three were predicted damaging the constitutive splice site. Using SpliceFinder to analyze aCML samples, these three novel splicing variants impacting the canonical AG/GT splice sites were identified for pt. 5, pt. 6, and pt. 7 (Table 1). All mutations were confirmed by Sanger method. In the other aCML patients (pt. 1, pt. 2, pt. 3, pt. 4, pt. 8) no splicing variants were found.

In pt. 5 we identified a splicing mutation near the 3’ acceptor splice site at position −1 in the intron 5 between exons 5 and 6 of the *SMAD9* (OMIM#603295) gene (NM_001127217.2:c.1004-1C>T), causing a loss of acceptor site. Despite the very high frequency of mutation 52% (Table 1, Fig. S4B and Table S2) the presence of a low gene expression (FPKM = 0.379) prevented us from building a reliable splicing map. The mean expression value in SMAD9-unmutated aCML samples was 0.538 FPKM.

**Figure 4:**
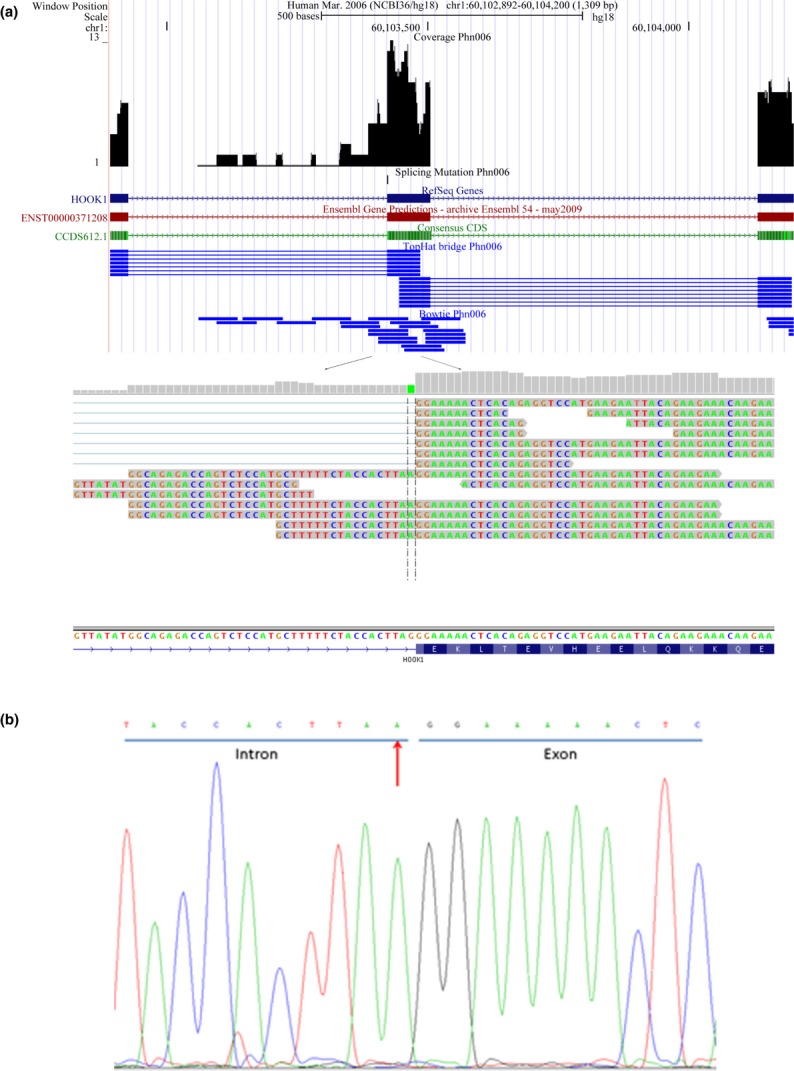
(a) RNA-seq read coverage of *HOOK1* (NM_015888) in Ph-006. RNA-seq showed seven junction reads between exon 17 and exon 18 (58% wild-type sequence) and five reads mapping on the acceptor site (42% intron retention) and carrying the splicing mutation. (b) Sanger sequencing of the aberrant *HOOK1* product showed the presence of the mutated base (adenine) and the absence of the wild-type guanine in the retained intron sequence meaning that the intron retention is exclusively caused by the mutation.

In pt. 6 the sequencing analysis identified a splicing point mutation near the 3’ acceptor splice site at position −1 in the intron between exons 17 and 18 of *HOOK1* (OMIM# 607820) gene (NM_015888.4:c.1662-1G>A) with a frequency of 47% (Table 1, Fig. S5A and Table S2). SpliceFinder analysis predicted the loss of a physiologic acceptor site (Table 2). RNA-Seq analysis was able to detect the intron mutation in five reads spanning the intron–exon junction confirming the presence of two splicing profiles: the wild-type mRNA and the altered *HOOK1* mRNA caused by a loss of constitutive acceptor splice site resulting in an intron retention. RNA-Seq analysis showed the presence of intron retention in 42% of the reads mapping the splicing junction (Fig. 4) and the splicing mutation resulting in a frameshift. Overall, the *HOOK1* expression level (3.767 FPKM, 51st percentile in pt. 6) was not different in pt. 6 compared with the other aCML samples (mean 4.518 FPKM, 55th percentile). Although other aCML cases showed reads mapping in intron 17, corresponding to the annotated processed transcript ENST0000046680 (Fig. S5B), the presence of the mutation and the absence of the wild-type guanine in the retained intron sequences indicate that in pt. 6 the *HOOK1* mutation is able to shift the equilibrium in favor of the intron retention splicing variant (Fig. 4a). Sequencing of the amplicon confirmed the presence of the heterozygous somatic variant and absence of the wild-type nucleotide, as expected (Fig. 4b).

The third somatic mutation affected position +5 (NM_001372.3:c.10242+5G>A) of the donor splice site of *DNAH9* (OMIM# 603330) intron 52 in pt. 7. The frequency of this variant in whole-exome sequencing analysis was 51% (Fig. S4C, Table S2). SpliceFinder analysis predicted a loss of donor site (Table 2), however, RNA-Seq analysis showed no expression of DNAH9 gene and very low mean expression value in other aCML patients (0 FPKM in pt. 7 and mean expression value of 0.019 FPKM in others aCML patients).

## Discussion

Several studies of leukemia and solid tumors focused the analysis on coding regions to find driver mutations (Stratton et al. 2009; Morin et al. 2010; Papaemmanuil et al. 2011; Yan et al. 2011; Landau et al. 2013; Piazza et al. 2013). In this study, we demonstrate the potential of whole-exome sequencing coupled with RNA-Seq for the identification and validation of splicing site mutations in cancer genomes. This combined approach, based on the identification of splicing sites mutations by exome sequencing and subsequent validation of abnormal transcripts with RNA-Seq, allows the implementation of a high-throughput pipeline for the selection of the functional splicing variants that are present in a cancer genome. Moreover, the combined use of exome sequencing and RNA-Seq allows to couple the identification of splicing variants to other analyses that are critical to thoroughly define the genomic landscape of cancer and leukemias, such as the identification of somatic variants in the coding regions (Larson et al. 2012; Roth et al. 2012; Cibulskis et al. 2013), the copy number and LOH analyses (Love et al. 2011; Sathirapongsasuti et al. 2011; Koboldt et al. 2012), the fusion detection (Sboner et al. 2010; Li et al. 2011; McPherson et al. 2011; Piazza et al. 2012) and the transcriptional analyses (Mortazavi et al. 2008; Trapnell et al. 2010; Garber et al. 2011; Tarazona et al. 2011).

By using our pipeline, we were able to identify a previously unreported homozygous somatic variant in a colon cancer sample predicted to affect *APC* splicing and six novel somatic mutations with a predicted effect on splicing in leukemic samples.

*APC* is a well-known target during the early stages of colon cancer: truncation of the APC protein occurs in >80% of colorectal cancers (CRC) (Rowan et al. 2000) and it is associated with the initial stages of oncogenic transformation (Kinzler and Vogelstein 1997; Jones et al. 2008), which suggests a functional role for our newly identified splicing variant. The c.1312+5G>A variation in *APC* was absent from dbSNP and COSMIC (http://www.sanger.ac.uk/genetics/CGP/cosmic/) databases. COSMIC has cataloged many confirmed somatic variations in *APC*, coding and intronic mutations (0.6%). Of them, the transversion (c.1312+4T>G) and the transition (c.1312+2T>C) affected, respectively, position +4 (chr5:112,182,943-112,182,944, genomic position NCBI36) (Vasovcak et al. 2011) and position +2 (112,182,941-112,182,942, genomic position NCBI36) (Nishisho et al. 1991) near the donor splice site of intron 10. The transition was reported as homozygous in (Miyoshi et al. 1992).

SpliceFinder identified heterozygous splicing mutations in the conserved regions of the *GNAQ*, *ABCC3*, *KLHDC1* genes in CML and *HOOK1*, *SMAD9*, *DNAH9* genes in aCML patients. All the somatic mutations were confirmed by Sanger method. None of these intronic mutations has previously been annotated in dbSNP and implicated in cancer development (COSMIC). It is notable that most of the variants are clustered in conserved regions within the consensus splicing sequences, thus impacting the canonical AG/GT splice sites similarly to what is already observed in *APC*. As most mutations will result in frameshift or in premature termination of protein synthesis, it is likely that they will have a deleterious effect on protein function, although direct experimental validation of the biological activity of the mutated protein has not been determined. It is also possible that certain nontranslated alternative transcripts may play a role in gene regulation (Lewis et al. 2003; Sorek et al. 2004; Skandalis et al. 2010).

In our study, RNA-Seq confirmed the power to detect nucleotide variations in transcribed regions as previously showed by several studies (Chepelev et al. 2009; Cirulli et al. 2010) even revealing the mutation in intron–exon junction for *HOOK1* gene, and the ability for the characterization of alternative splicing patterns (Trapnell et al. 2009; Ameur et al. 2010). By using RNA-Seq sequencing information on junction reads of the expressed genes, we were able to confirm whether or not a mutation in the intronic splice sites resulted in an real effect on RNA splicing. We reconstructed the splicing of *GNAQ*, *ABCC3,* and *HOOK1* and we confirmed the splicing prediction analysis based on genomic DNA analysis. The expression level of the aberrant and the wild-type transcripts was similar for *GNAQ* and *HOOK1*, but lower in *ABCC3*. A deeper RNA-Seq sequencing will be needed to thoroughly assess whether the mutant allele expression is similar to the wild-type one. A lower expression level of the abnormal mRNA, as detected in *ABCC3*, may be caused by the activation of the nonsense-mediated mRNA decay (NMD) pathway that selectively and rapidly degrades the transcripts harboring mutations and premature termination codons (Johnson et al. 2012).

We evaluated the presence of aberrant splicing in *GNAQ* gene caused by NM_002072.2:c.735+1C>T mutation by RT-PCR. This analysis showed and confirmed the presence of two different isoforms in the leukemic sample compared to a single wild-type isoform in the paired remission sample; the longer isoform corresponding to the canonical one and the shorter one to the aberrant transcript. The Sanger sequence of the short transcript showed a frameshift splicing with loss of exon 5 in the mutant sequence.

The *GNAQ* (NM_002072.2:c.735+1C>T) variant has not been reported in literature while coding mutations were extensively described. The most frequent somatic mutation occurred in codon 209 in the *RAS*-like domain (COSMIC). Q209 can cause complete or partial loss of intrinsic GTPase activity, thereby locking the protein in a constitutively active form (Landis et al. 1989; Kalinec et al. 1992). In melanoma, Q209 resulted in constitutive activation of GNAQ leading to activation of the *MAPK* pathway (Van Raamsdonk et al. 2009). So far no activating mutations of GNAQ in leukemias have been reported. In our case the out-of-frame deletion resulted in a premature stop codon leading to the complete loss of the GNAQ GTPase domain.

The presence of intron retention in *HOOK1* caused by NM_015888.4:c.1662-1G>A mutation was functionally validated by demonstrating the exclusive presence of the mutated variant in the cDNA carrying the intron retention. Additional research is required to characterize the biological effect of the resulting aberrant mRNA. However, the NM_015888.4:c.1662-1G>A mutation has not been reported in literature. Moreover, so far COSMIC has cataloged 28 *HOOK1* somatic mutations in solid cancer tissues but none was previously found in leukemia. Among them, two splicing mutations affecting the donor splice site are associated with lung cancer.

On the basis of the weak expression, we did not consider further validation on *KLHDC1, SMAD9,* and *DNAH9* mutations. However, the absence of expression in the cell population under analysis does not exclude a functional role for that mutation. One of the critical features of aCML (and classical CML) is that even if the leukemic cells are no longer under the physiological control of the cell cycle, they are still able to differentiate almost normally from the leukemic hematopoietic stem cell (HSC) to the completely differentiated myeloid cell. This means that, even if in presence of “clonal” cancer cells, the cancer transcriptome profile is largely heterogeneous: a gene that is not expressed in the majority of the differentiated leukemic cells could still be expressed at high level in the rare leukemic HSC. Testing this hypothesis, however, goes beyond the scope of this work. Information about the role of the mutated genes and their impact in solid cancer or leukemia are obtained from the GeneCards (http://www.genecards.org/) and the GeneRanker (http://cbio.mskcc.org/tcga-generanker/index.jsp) databases (Table S3).

However, the variants identified here should be subjected to extensive analyses to assess if the aberrant transcript translation product can be functional or nonfunctional (Melamud and Moult 2009), to dissect their potential phenotypic effects and to assess the clinical significance of these variants in leukemias.

In conclusion, we showed the applicability of SpliceFinder as a methodology to identify novel splicing variants and to select those true-positive intronic variants that are predicted to affect RNA splicing. The combination of DNA analysis and gene expression profiling provides a powerful approach to identify new alternative splicing events. This knowledge will form the basis for better understanding the nature of cancer and to increase the likelihood of identifying functional mutations in patients (Grossmann et al. 2011).

## Supporting Information

**Figure S1.** *APC* (NM_000038.4:c.1312+5G>A) splicing mutation detected by whole-exome sequencing. (A) Read coverage in colon cancer sample and (B) in paired peripheral blood sample.

**Figure S2.** Skipping of exon 5 of *GNAQ* in Ph+001 sample resulted from RNA-Seq data. (A) RNA-seq read coverage in UCSC panel. RNA-Seq showed 28 junction reads between exons 4 and 6 resulting in 78% of mutant sequence and five junction reads between exon 5 and exon 6 and three junction reads between exon 4 and exon 5 resulting in 22% of wild-type sequence. (B) *GNAQ* splicing site in the other CML patients.

**Figure S3.** (A) *ABCC**3* (NM_003786.3:c.1783-1G>A) splicing mutation near the 3’ acceptor splice site at position −1 in the intron between exons 13 and 14 in UCSC panel and somatic mutation frequency of 51% in IGV panel. Below, the Sanger validation. (B) RNA-Seq read coverage of ABCC3 (NM_002072) in Ph+004. RNA-Seq showed 11 junction reads between exon 13 and exon 14 (58% wild-type sequence), seven reads mapping in intron 13 (37% intron retention) and one read (5%) mapping 5 bases within the exon 14. (C) ABCC3 splicing site in the other CML and aCML patients.

**Figure S4.** Whole-exome and Sanger sequencing of *KLHDC**1,* *SMAD**9,* and *DNAH**9* somatic mutations. (A) *KLHDC**1* (NM_172193.1:c.568-2A>G) splicing mutation near the 3’ acceptor splice site at position −2 in the intron between exons 6 and 7 with a frequency of 52%. Below, the Sanger validation. (B) *SMAD**9* (NM_001127217.2:c.1004-1C>T) splicing mutation near the 3’ acceptor splice site at position −1 in the intron between exons 5 and 6 with a frequency of 52%. Below, the Sanger validation. (C) *DNAH**9* (NM_001372.3:c.10242+5G>A) splicing mutation near the 5’ donor site at position +5 in the intron between exons 52 and 53 with a frequency of 51%.

**Figure S5.** (A) *HOOK**1* (NM_015888.4:c.1662-1G>A) splicing mutation near the 3’ acceptor splice site at position −1 in the intron between exons 17 and 18 in UCSC panel and somatic mutation frequency of 47%. Below, the Sanger validation. (B) *HOOK**1* splice site in aCML dataset.

**Figure S6.** (A–F)Whole-exome sequencing of wild-type *GNAQ**,* *ABCC**3*, *KLHDC**1,* *SMAD**9,* *HOOK**1,* and *DNAH**9* genes in normal lymphocytes, respectively, in patients Ph+001, Ph+004, Ph+005, Ph-005, Ph-006, and Ph-007. (G) Whole-exome sequencing of mutated *HOOK**1* in Ph-006.

**Table S1.** Functional annotation summary of somatic variants in CML (A) and aCML (B) patients by ANNOVAR. Exons, variant overlapping a coding exon; Splicing, variant within 20 bp from a splicing junction; UTRs, variant overlapping a 5’ untranslated region or a 3’ untranslated region; Introns, variant overlapping an intron; Intergenic, variant in an intergenic region; ncRNA, variant overlapping a noncoding transcript.

**Table S2.** PCR and sequencing primers used to validate the somatic mutations identified by whole-exome sequencing.

**Table S3.** Proteins encoded by six splicing mutated genes in CML or aCML and correlation with other cancers. Data taken from GeneCards, COSMIC, and GeneRanker databases. The “Cancer” column indicates the cancer tissues previously confirmed somatically mutated from COSMIC database. *AML, acute myeloid leukemia; CLL, chronic lymphocytic leukemia-small lymphocytic lymphoma; aCML.

## References

[b1] Adzhubei IA, Schmidt S, Peshkin L, Ramensky VE, Gerasimova A, Bork P (2010). A method and server for predicting damaging missense mutations. Nat. Methods.

[b2] Ameur A, Wetterbom A, Feuk L, Gyllensten U (2010). Global and unbiased detection of splice junctions from RNA-seq data. Genome Biol.

[b3] Asselta R, Montefusco MC, Duga S, Malcovati M, Peyvandi F, Mannucci PM (2003). Severe factor V deficiency: exon skipping in the factor V gene causing a partial deletion of the C1 domain. J. Thromb. Haemost.

[b4] Blankenberg D, Coraor G, Von Kuster N, Ananda G, Lazarus R, Mangan M (2010). Galaxy: a web-based genome analysis tool for experimentalists. Curr. Protoc. Mol. Biol.

[b5] Brunak S, Engelbrecht J, Knudsen S (1991). Prediction of human mRNA donor and acceptor sites from the DNA sequence. J. Mol. Biol.

[b6] Chen X, Truong TT, Weaver J, Bove BA, Cattie K, Armstrong BA (2006). Intronic alterations in BRCA1 and BRCA2: effect on mRNA splicing fidelity and expression. Hum. Mutat.

[b7] Chepelev I, Wei G, Tang Q, Zhao K (2009). Detection of single nucleotide variations in expressed exons of the human genome using RNA-Seq. Nucleic Acids Res.

[b8] Chun S, Fay JC (2009). Identification of deleterious mutations within three human genomes. Genome Res.

[b9] Cibulskis K, Lawrence MS, Carter SL, Sivachenko A, Jaffe D, Sougnez C (2013). Sensitive detection of somatic point mutations in impure and heterogeneous cancer samples. Nat. Biotechnol.

[b10] Cirulli ET, Singh A, Shianna KV, Ge D, Smith JP, Maia JM (2010). Screening the human exome: a comparison of whole genome and whole transcriptome sequencing. Genome Biol.

[b11] Ewing B, Green P (1998). Base-calling of automated sequencer traces using phred. II. Error probabilities. Genome Res.

[b12] Fackenthal JD, Godley LA (2008). Aberrant RNA splicing and its functional consequences in cancer cells. Dis. Model Mech.

[b13] Forbes SA, Tang G, Bindal N, Bamford S, Dawson E, Cole C (2010). COSMIC (the catalogue of somatic mutations in cancer): a resource to investigate acquired mutations in human cancer. Nucleic Acids Res.

[b14] Garber M, Grabherr MG, Guttman M, Trapnell C (2011). Computational methods for transcriptome annotation and quantification using RNA-seq. Nat. Methods.

[b15] Giardine B, Riemer C, Hardison RC, Burhans R, Elnitski L, Shah P (2005). Galaxy: a platform for interactive large-scale genome analysis. Genome Res.

[b16] Goecks J, Nekrutenko A, Taylor J (2010). Galaxy: a comprehensive approach for supporting accessible, reproducible, and transparent computational research in the life sciences. Genome Biol.

[b17] Grossmann V, Tiacci E, Holmes AB, Kohlmann A, Martelli MP, Kern W (2011). Whole-exome sequencing identifies somatic mutations of BCOR in acute myeloid leukemia with normal karyotype. Blood.

[b18] Gutierrez-Enriquez S, Coderch V, Masas M, Balmana J, Diez O (2009). The variants BRCA1 IVS6-1G>A and BRCA2 IVS15+1G>A lead to aberrant splicing of the transcripts. Breast Cancer Res. Treat.

[b19] Hand DJ, Yu KM (2001). Idiot's Bayes—not so stupid after all?. Int. Stat. Rev.

[b20] He C, Zhou F, Zuo Z, Cheng H, Zhou R (2009). A global view of cancer-specific transcript variants by subtractive transcriptome-wide analysis. PLoS One.

[b21] Hebsgaard SM, Korning PG, Tolstrup N, Engelbrecht J, Rouze P, Brunak S (1996). Splice site prediction in Arabidopsis thaliana pre-mRNA by combining local and global sequence information. Nucleic Acids Res.

[b22] Houdayer C, Dehainault C, Mattler C, Michaux D, Caux-Moncoutier V, Pages-Berhouet S (2008). Evaluation of in silico splice tools for decision-making in molecular diagnosis. Hum. Mutat.

[b23] Johnson JK, Waddell N, Chenevix-Trench G (2012). The application of nonsense-mediated mRNA decay inhibition to the identification of breast cancer susceptibility genes. BMC Cancer.

[b24] Jones S, Chen WD, Parmigiani G, Diehl F, Beerenwinkel N, Antal T (2008). Comparative lesion sequencing provides insights into tumor evolution. Proc. Natl. Acad. Sci. USA.

[b25] Kalinec G, Nazarali AJ, Hermouet S, Xu N, Gutkind JS (1992). Mutated alpha subunit of the Gq protein induces malignant transformation in NIH 3T3 cells. Mol. Cell. Biol.

[b26] Kinzler KW, Vogelstein B (1997). Cancer-susceptibility genes. Gatekeepers and caretakers. Nature.

[b27] Koboldt DC, Zhang Q, Larson DE, Shen D, McLellan MD, Lin L (2012). VarScan 2: somatic mutation and copy number alteration discovery in cancer by exome sequencing. Genome Res.

[b28] Krawczak M, Reiss J, Cooper DN (1992). The mutational spectrum of single base-pair substitutions in mRNA splice junctions of human genes: causes and consequences. Hum. Genet.

[b29] Kumar P, Henikoff S, Ng PC (2009). Predicting the effects of coding nonsynonymous variants on protein function using the SIFT algorithm. Nat. Protoc.

[b30] Landau DA, Carter SL, Stojanov P, McKenna A, Stevenson K, Lawrence MS (2013). Evolution and impact of subclonal mutations in chronic lymphocytic leukemia. Cell.

[b31] Landis CA, Masters SB, Spada A, Pace AM, Bourne HR, Vallar L (1989). GTPase inhibiting mutations activate the alpha chain of Gs and stimulate adenylyl cyclase in human pituitary tumours. Nature.

[b32] Langmead B, Trapnell C, Pop M, Salzberg SL (2009). Ultrafast and memory-efficient alignment of short DNA sequences to the human genome. Genome Biol.

[b33] Larson DE, Harris CC, Chen K, Koboldt DC, Abbott TE, Dooling DJ (2012). SomaticSniper: identification of somatic point mutations in whole genome sequencing data. Bioinformatics.

[b34] Lewis BP, Green RE, Brenner SE (2003). Evidence for the widespread coupling of alternative splicing and nonsense-mediated mRNA decay in humans. Proc. Natl. Acad. Sci. USA.

[b35] Li H, Durbin R (2009). Fast and accurate short read alignment with Burrows-Wheeler transform. Bioinformatics.

[b36] Li H, Handsaker B, Wysoker A, Fennell T, Ruan J, Homer N (2009). The sequence alignment/map format and SAMtools. Bioinformatics.

[b37] Li Y, Chien J, Smith DI, Ma J (2011). FusionHunter: identifying fusion transcripts in cancer using paired-end RNA-seq. Bioinformatics.

[b38] Liu X, Jian X, Boerwinkle E (2011). dbNSFP: a lightweight database of human nonsynonymous SNPs and their functional predictions. Hum. Mutat.

[b39] Love MI, Mysickova A, Sun R, Kalscheuer V, Vingron M, Haas SA (2011). Modeling read counts for CNV detection in exome sequencing data. Stat. Appl. Genet. Mol. Biol.

[b40] McPherson A, Hormozdiari F, Zayed A, Giuliany R, Ha G, Sun MG (2011). deFuse: an algorithm for gene fusion discovery in tumor RNA-Seq data. PLoS Comput. Biol.

[b41] Melamud E, Moult J (2009). Structural implication of splicing stochastics. Nucleic Acids Res.

[b42] Miyoshi Y, Nagase H, Ando H, Horii A, Ichii S, Nakatsuru S (1992). Somatic mutations of the *APC* gene in colorectal tumors: mutation cluster region in the *APC* gene. Hum. Mol. Genet.

[b43] Morin RD, Johnson NA, Severson TM, Mungall AJ, An J, Goya R (2010). Somatic mutations altering EZH2 (Tyr641) in follicular and diffuse large B-cell lymphomas of germinal-center origin. Nat. Genet.

[b44] Mortazavi A, Williams BA, McCue K, Schaeffer L, Wold B (2008). Mapping and quantifying mammalian transcriptomes by RNA-Seq. Nat. Methods.

[b45] Nishisho I, Nakamura Y, Miyoshi Y, Miki Y, Ando H, Horii A (1991). Mutations of chromosome 5q21 genes in FAP and colorectal cancer patients. Science.

[b46] Papaemmanuil E, Cazzola M, Boultwood J, Malcovati L, Vyas P, Bowen D (2011). Somatic SF3B1 mutation in myelodysplasia with ring sideroblasts. N. Engl. J. Med.

[b47] Piazza R, Pirola A, Spinelli R, Valletta S, Redaelli S, Magistroni V (2012). FusionAnalyser: a new graphical, event-driven tool for fusion rearrangements discovery. Nucleic Acids Res.

[b48] Piazza R, Valletta S, Winkelmann N, Redaelli S, Spinelli R, Pirola A (2013). Recurrent SETBP1 mutations in atypical chronic myeloid leukemia. Nat. Genet.

[b49] Ramensky V, Bork P, Sunyaev S (2002). Human non-synonymous SNPs: server and survey. Nucleic Acids Res.

[b50] Reese MG, Eeckman FH, Kulp D, Haussler D (1997). Improved splice site detection in Genie. J. Comput. Biol.

[b51] Robinson JT, Thorvaldsdottir H, Winckler W, Guttman M, Lander ES, Getz G (2011). Integrative genomics viewer. Nat. Biotechnol.

[b52] Roth A, Ding J, Morin R, Crisan A, Ha G, Giuliany R (2012). JointSNVMix: a probabilistic model for accurate detection of somatic mutations in normal/tumour paired next-generation sequencing data. Bioinformatics.

[b53] Rowan AJ, Lamlum H, Ilyas M, Wheeler J, Straub J, Papadopoulou A (2000). APC mutations in sporadic colorectal tumors: a mutational “hotspot” and interdependence of the “two hits”. Proc. Natl. Acad. Sci. USA.

[b54] Sathirapongsasuti JF, Lee H, Horst BA, Brunner G, Cochran AJ, Binder S (2011). Exome sequencing-based copy-number variation and loss of heterozygosity detection: ExomeCNV. Bioinformatics.

[b55] Sboner A, Habegger L, Pflueger D, Terry S, Chen DZ, Rozowsky JS (2010). FusionSeq: a modular framework for finding gene fusions by analyzing paired-end RNA-sequencing data. Genome Biol.

[b56] Schwarz JM, Rodelsperger C, Schuelke M, Seelow D (2010). MutationTaster evaluates disease-causing potential of sequence alterations. Nat. Methods.

[b57] Skandalis A, Frampton M, Seger J, Richards MH (2010). The adaptive significance of unproductive alternative splicing in primates. RNA.

[b58] Skotheim RI, Nees M (2007). Alternative splicing in cancer: noise, functional, or systematic?. Int. J. Biochem. Cell Biol.

[b59] Sorek R, Shamir R, Ast G (2004). How prevalent is functional alternative splicing in the human genome?. Trends Genet.

[b60] Stratton MR, Campbell PJ, Futreal PA (2009). The cancer genome. Nature.

[b61] Tarazona S, Garcia-Alcalde F, Dopazo J, Ferrer A, Conesa A (2011). Differential expression in RNA-seq: a matter of depth. Genome Res.

[b62] Trapnell C, Pachter L, Salzberg SL (2009). TopHat: discovering splice junctions with RNA-Seq. Bioinformatics.

[b63] Trapnell C, Williams BA, Pertea G, Mortazavi A, Kwan G, van Baren MJ (2010). Transcript assembly and quantification by RNA-Seq reveals unannotated transcripts and isoform switching during cell differentiation. Nat. Biotechnol.

[b64] Van Raamsdonk CD, Bezrookove V, Green G, Bauer J, Gaugler L, O'Brien JM (2009). Frequent somatic mutations of GNAQ in uveal melanoma and blue naevi. Nature.

[b65] Vardiman JW, Thiele J, Arber DA, Brunning RD, Borowitz MJ, Porwit A (2009). The 2008 revision of the World Health Organization (WHO) classification of myeloid neoplasms and acute leukemia: rationale and important changes. Blood.

[b66] Vasovcak P, Pavlikova K, Sedlacek Z, Skapa P, Kouda M, Hoch J (2011). Molecular genetic analysis of 103 sporadic colorectal tumours in Czech patients. PLoS One.

[b67] Vreeswijk MP, Kraan JN, Vink HM, van der Klift GR, Cornelisse CJ, Wijnen JT (2009). Intronic variants in BRCA1 and BRCA2 that affect RNA splicing can be reliably selected by splice-site prediction programs. Hum. Mutat.

[b68] Wang GS, Cooper TA (2007). Splicing in disease: disruption of the splicing code and the decoding machinery. Nat. Rev. Genet.

[b69] Wang K, Li M, Hakonarson H (2010). ANNOVAR: functional annotation of genetic variants from high-throughput sequencing data. Nucleic Acids Res.

[b70] Xu G, Deng N, Zhao Z, Judeh T, Flemington E, Zhu D (2011). SAMMate: a GUI tool for processing short read alignments in SAM/BAM format. Source Code Biol. Med.

[b71] Yan XJ, Xu J, Gu ZH, Pan CM, Lu G, Shen Y (2011). Exome sequencing identifies somatic mutations of DNA methyltransferase gene DNMT3A in acute monocytic leukemia. Nat. Genet.

