# Identification of novel point mutations in splicing sites integrating whole-exome and RNA-seq data in myeloproliferative diseases

**DOI:** 10.1002/mgg3.77

**Published:** 2014-03-13

**Authors:** Roberta Spinelli, Alessandra Pirola, Sara Redaelli, Nitesh Sharma, Hima Raman, Simona Valletta, Vera Magistroni, Rocco Piazza, Carlo Gambacorti-Passerini

The original article to which this erratum refers was published in *Molecular Genetics and Genomic Medicine* 1(4):246–259 (DOI: 10.1002/mgg3.23).

In the published article, there are some errors in the authors' affiliations, which have been corrected in this erratum.

Below is the list of authors showing the corrected affiliations:

Roberta Spinelli^1^, Alessandra Pirola^1^, Sara Redaelli^1^, Nitesh Sharma^1^, Hima Raman^1^, Simona Valletta^1^, Vera Magistroni^1^, Rocco Piazza^1^,*, & Carlo Gambacorti-Passerini^1^,^2^,*

We regret these errors.

## Notes

1 Department of Health Sciences, University of Milano-Bicocca, Monza, Italy

2 Hematology and Clinical Research Unit, San Gerardo Hospital, Monza, Italy

* Rocco Piazza and Carlo Gambacorti-Passerini served as co-last authors

