# Supplementary figures and images for: Identification of novel point mutations in splicing sites integrating whole-exome and RNA-seq data in myeloproliferative diseases
Source: Mol Genet Genomic Med. 2013 Jul 7;1(4):246–59. doi: 10.1002/mgg3.23 (PMC3865592; doi:10.1002/mgg3.23)

Suppl. Fig. 1

A)

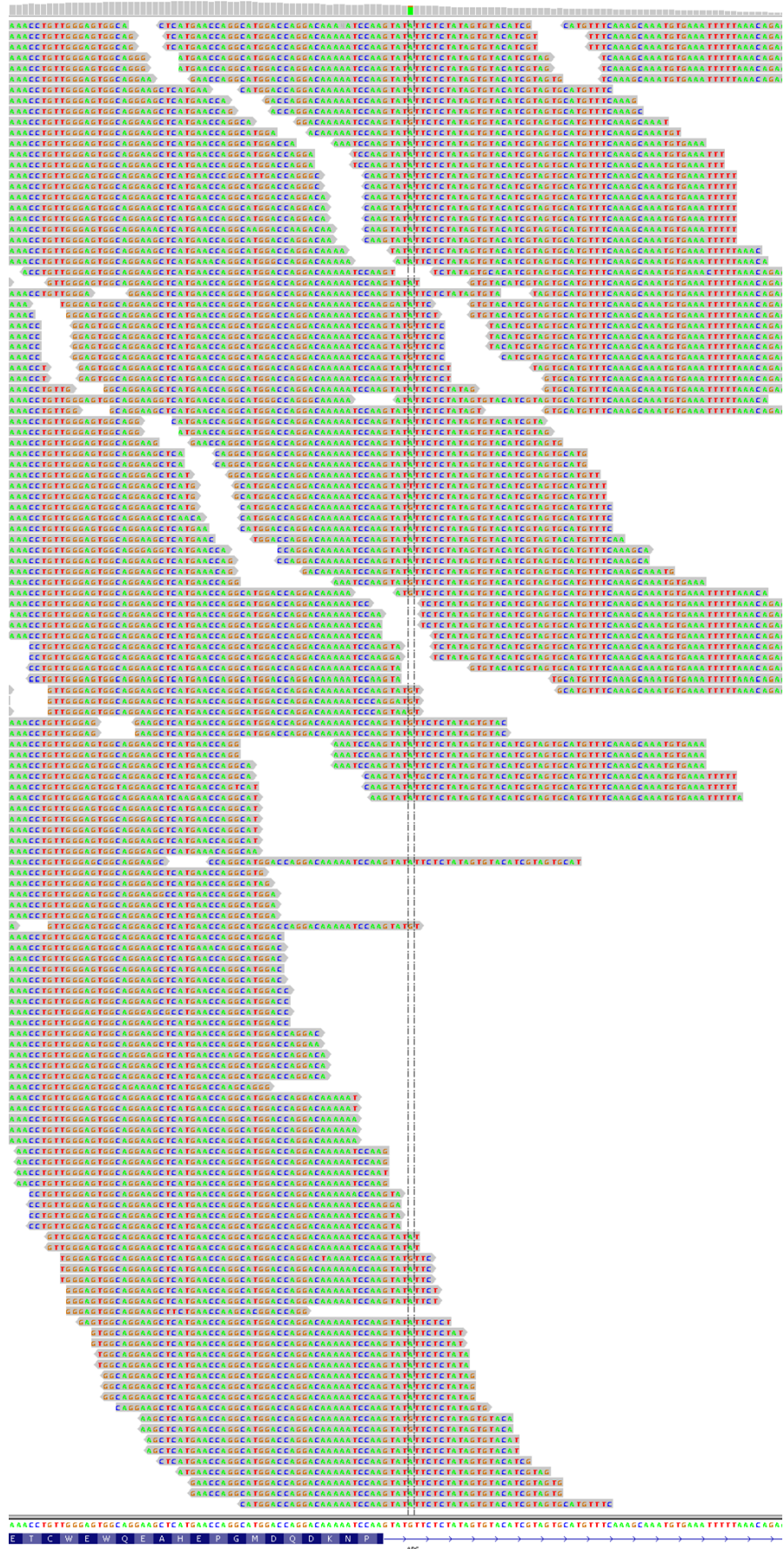

[illegible]

Supplement: Supplementary file 1 [file mgg30001-0246-SD1.pdf]

A)

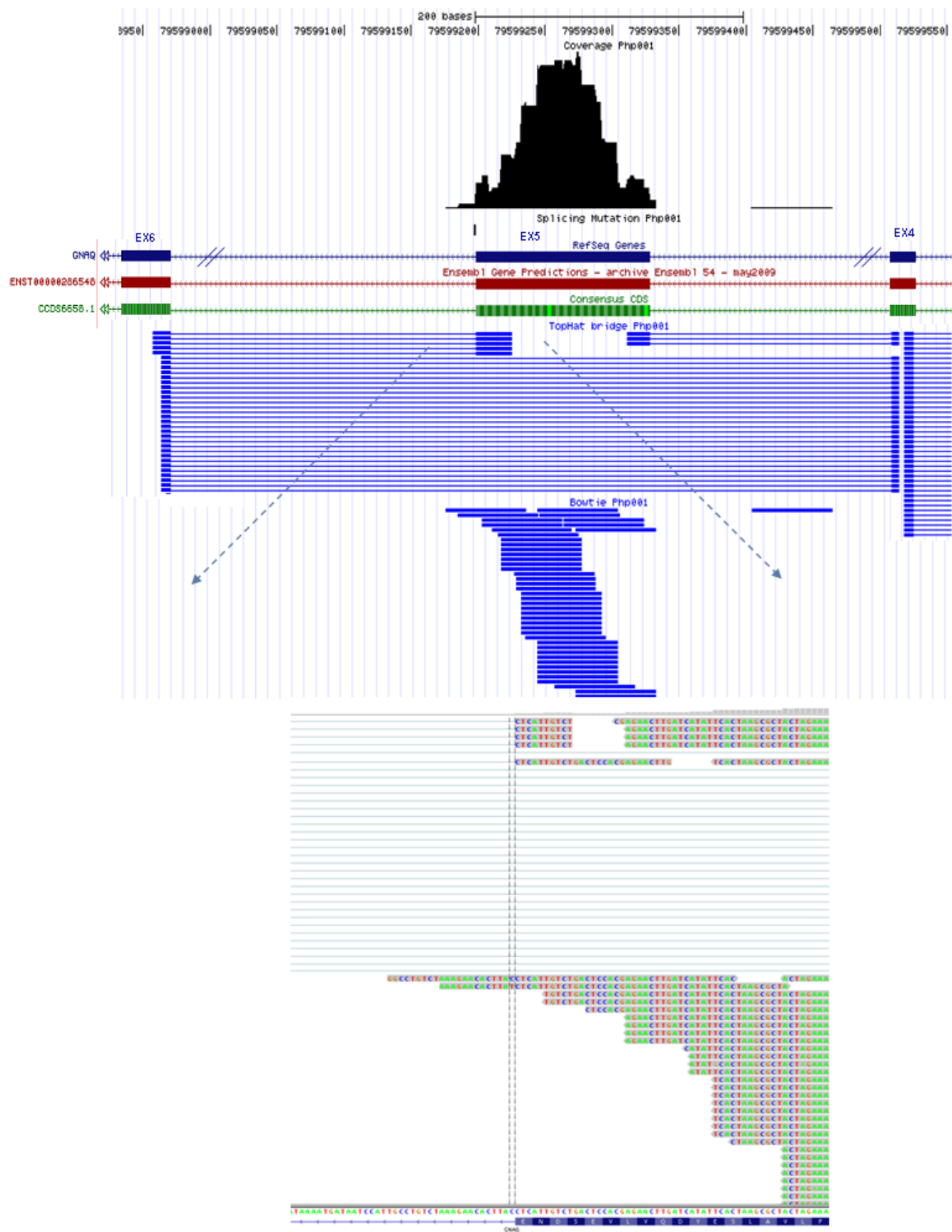

B)

Ph+002

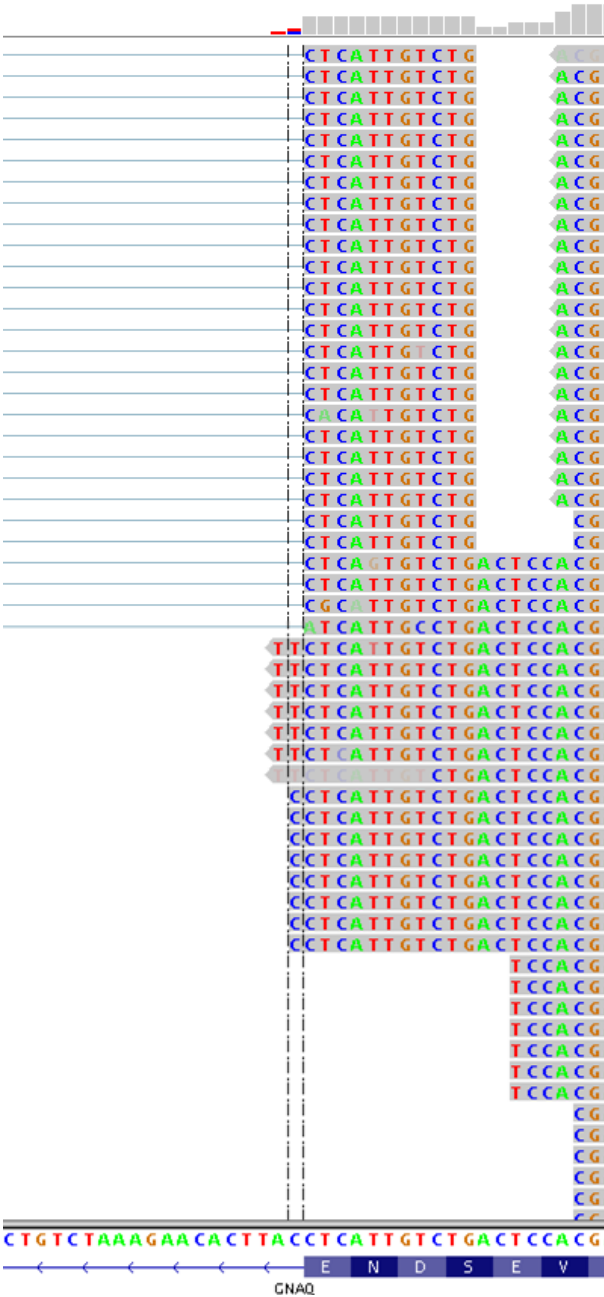

Ph+003

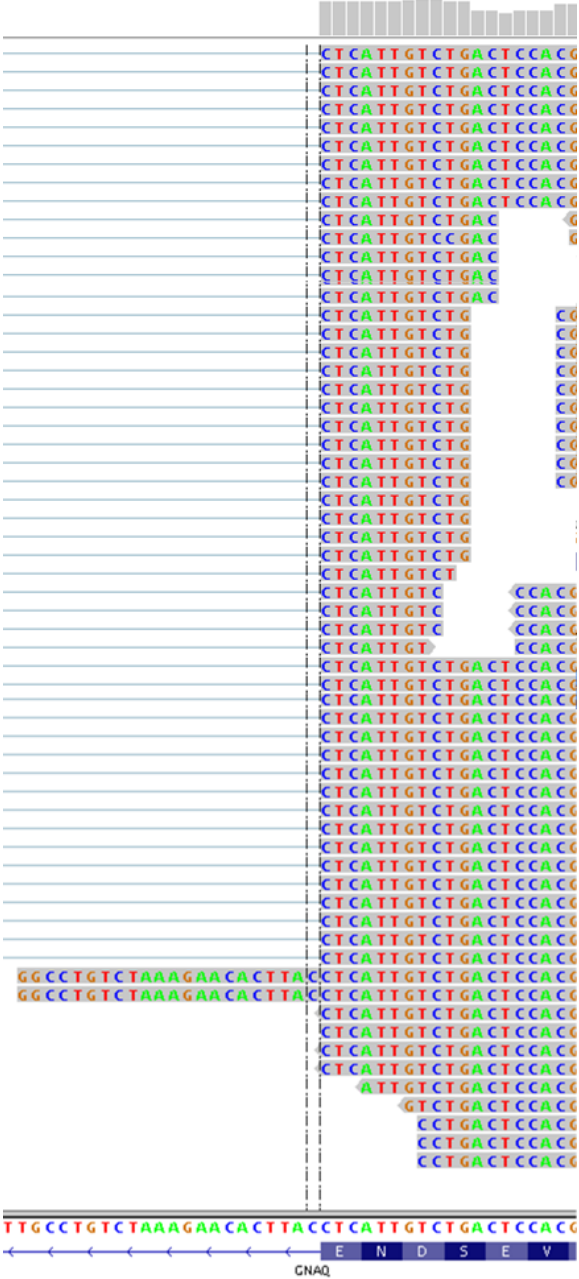

Ph+004

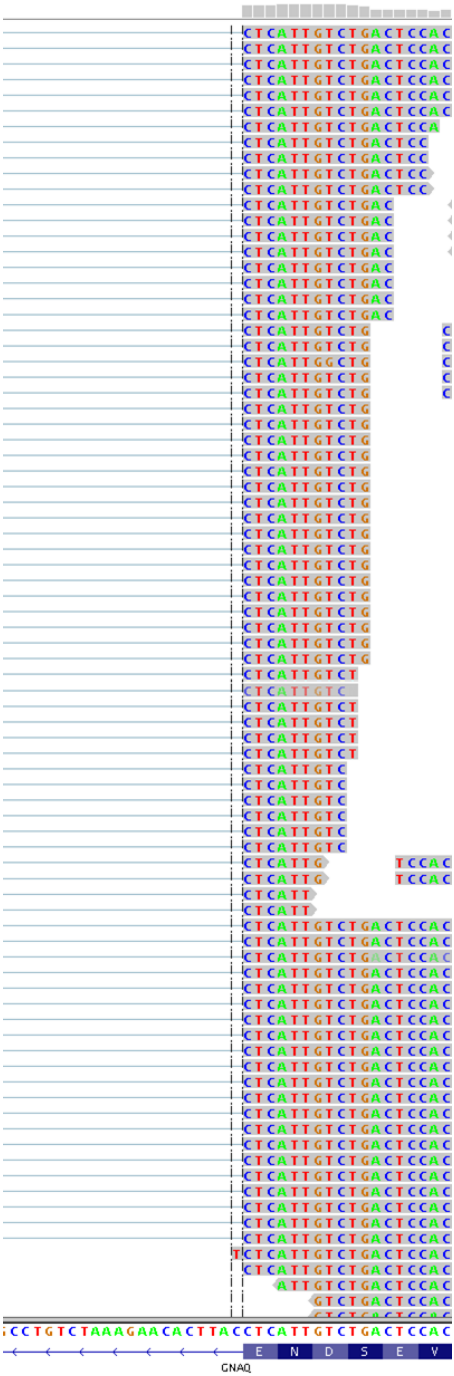

Ph+005

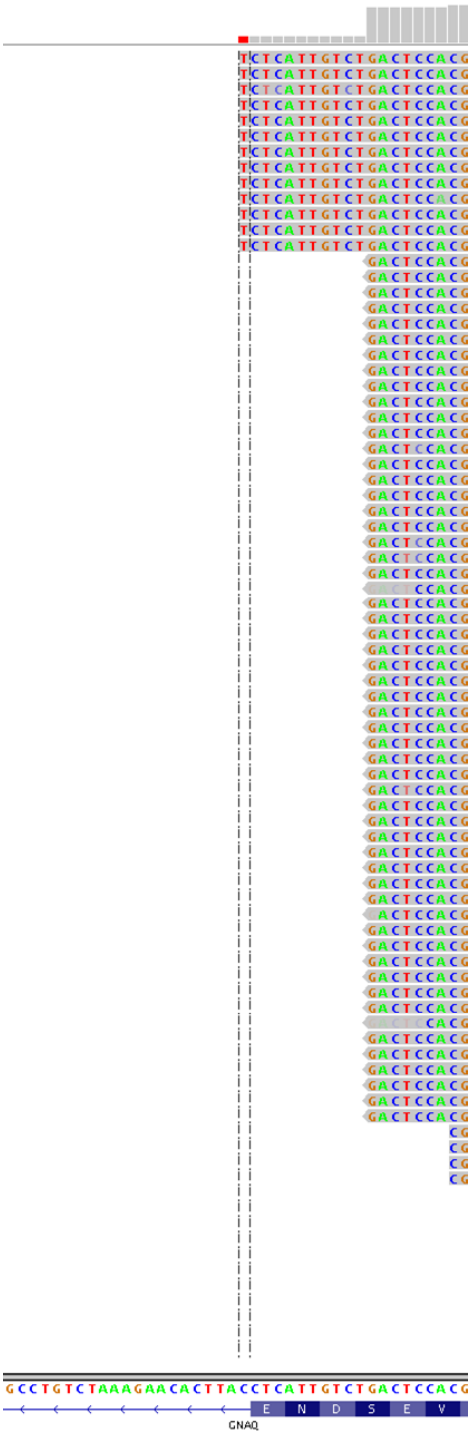

[illegible]

Supplement: Supplementary file 2 [file mgg30001-0246-SD2.pdf]

A)

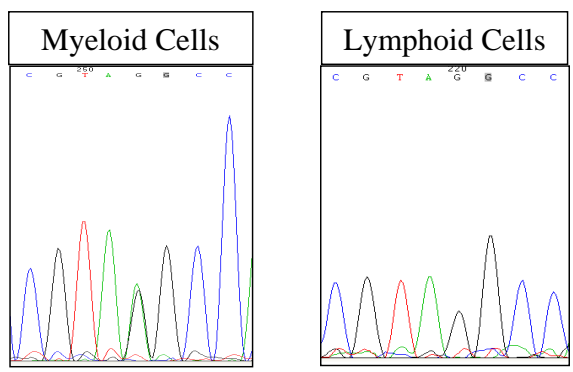

B)

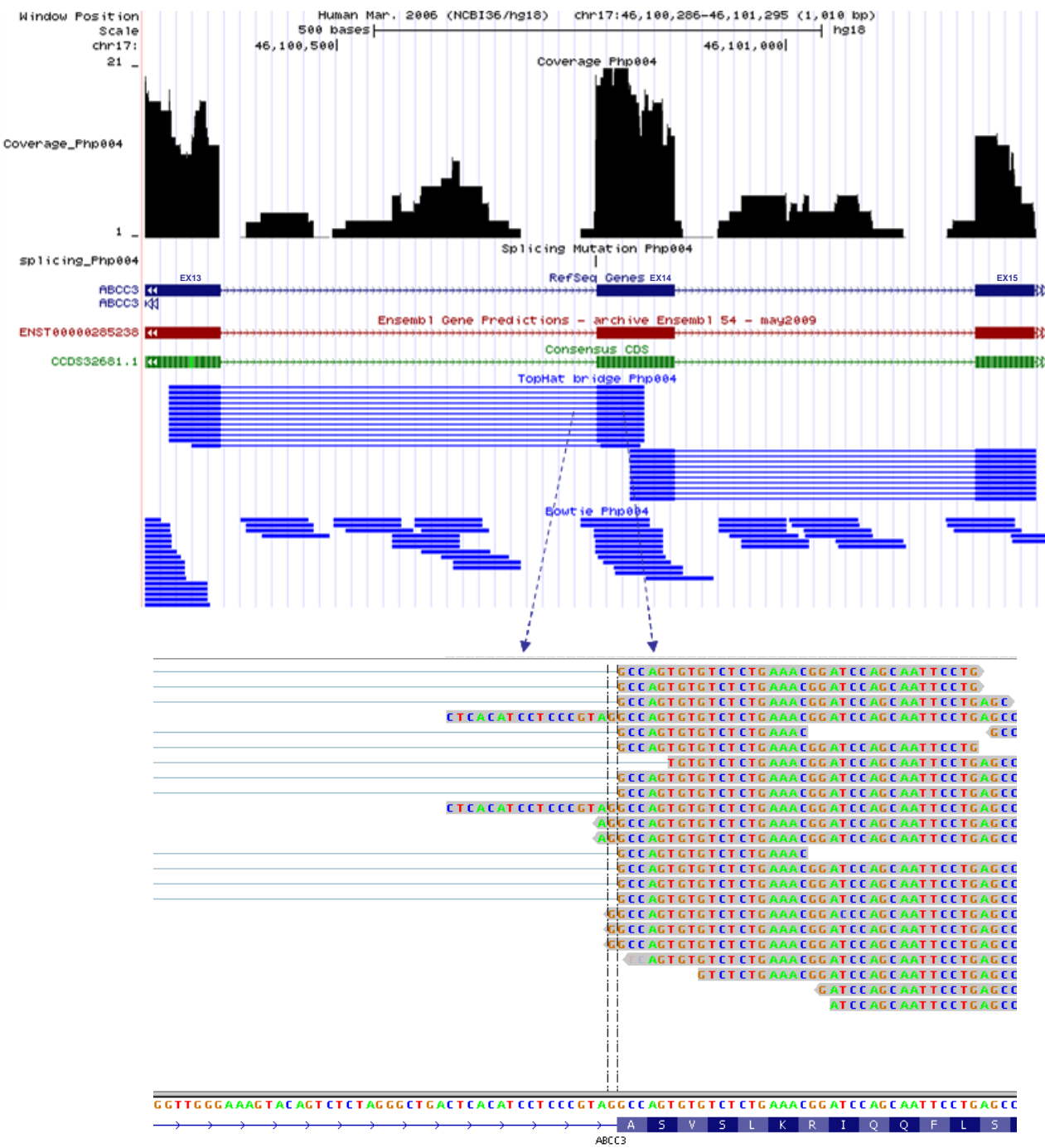

C) Ph+001

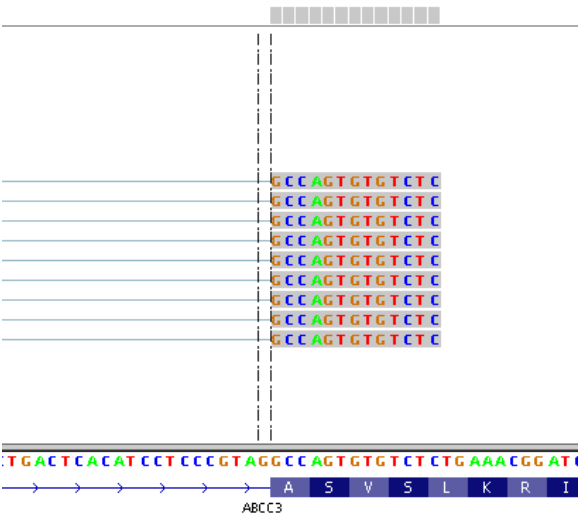

Ph+002

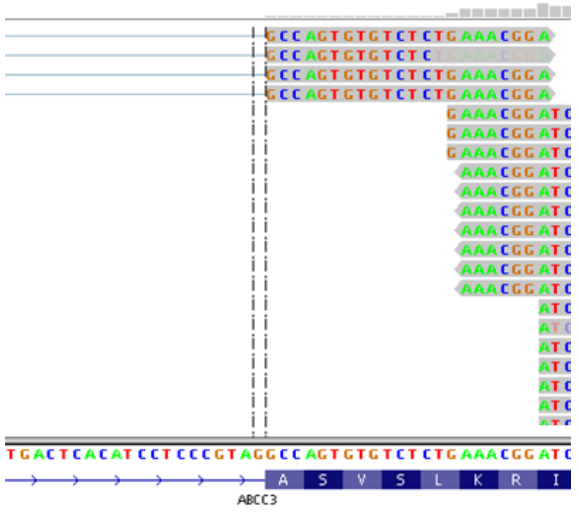

Ph+003

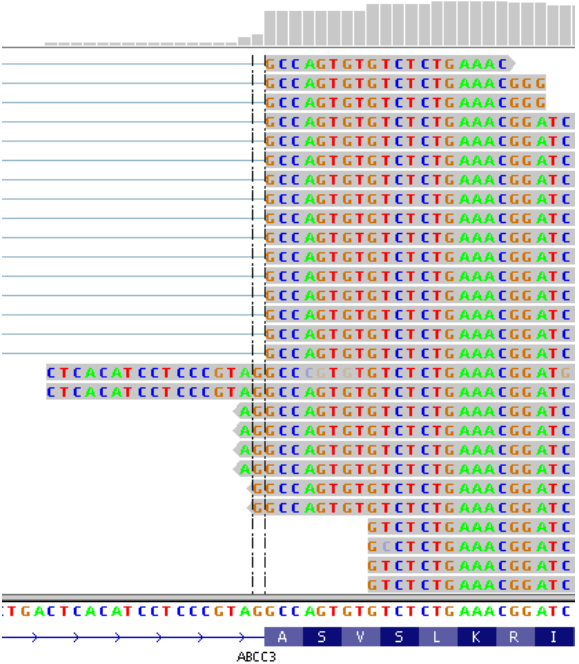

Ph+005

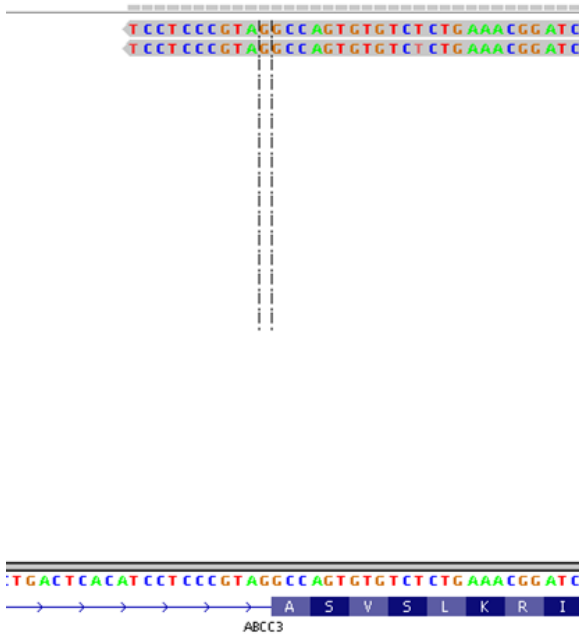

Ph+006

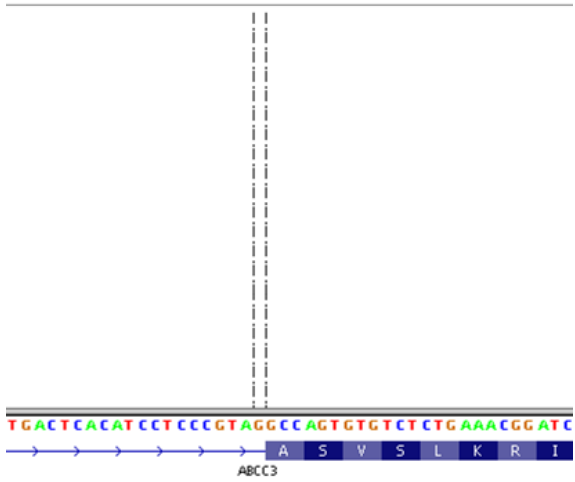

Ph+007

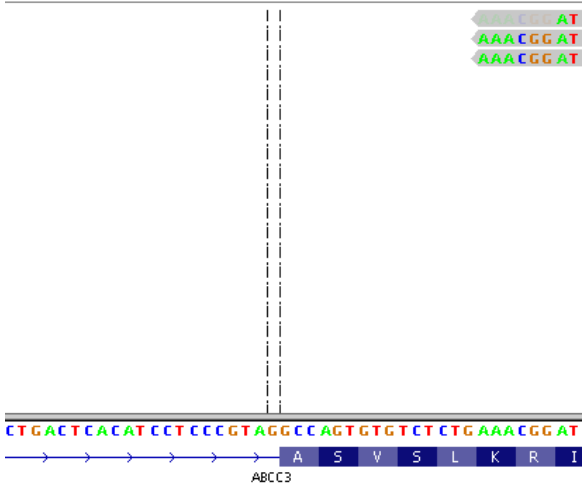

Ph+008

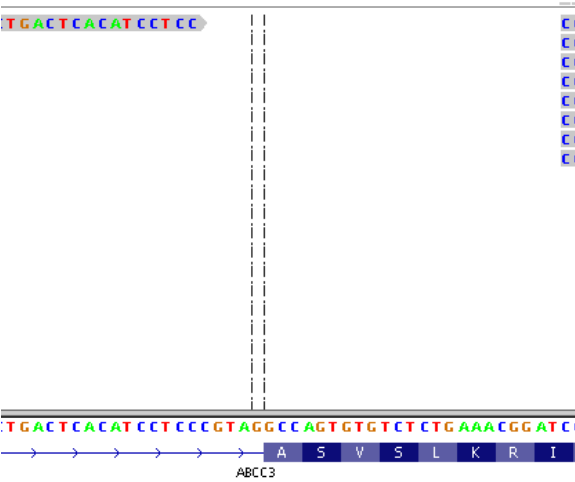

Ph-003

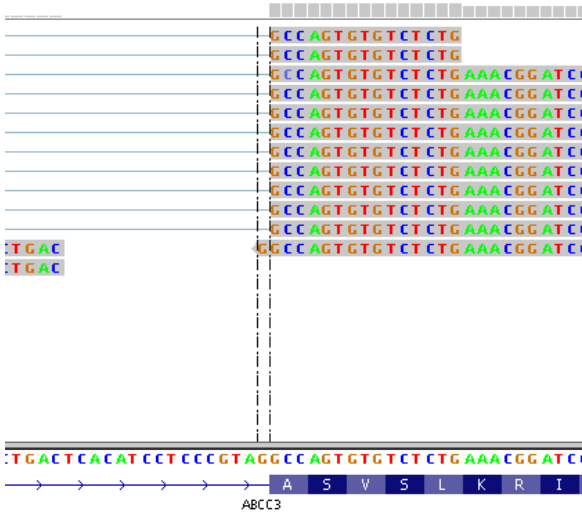

Supplement: Supplementary file 3 [file mgg30001-0246-SD3.pdf]

Suppl Fig. 4  
A)

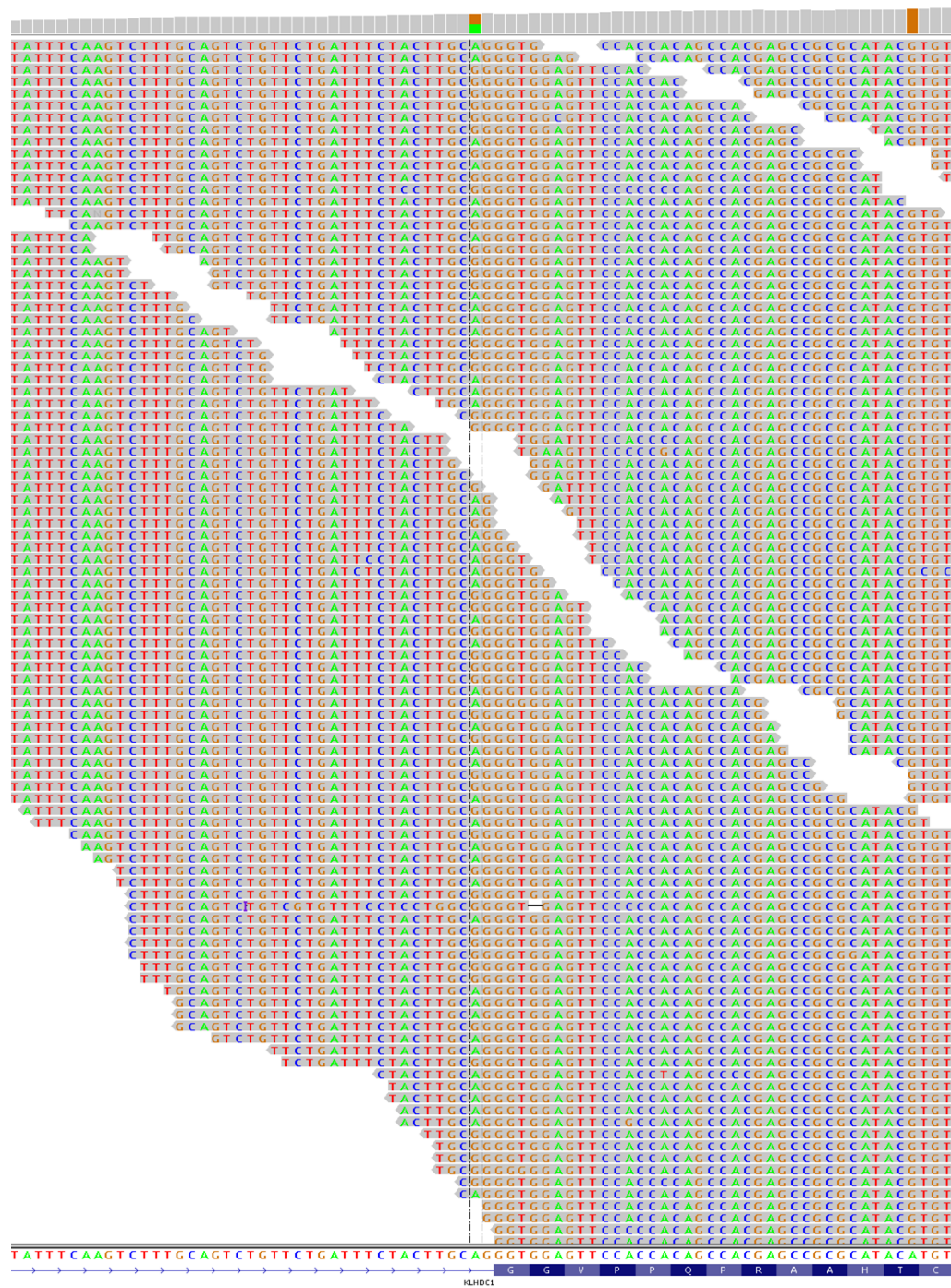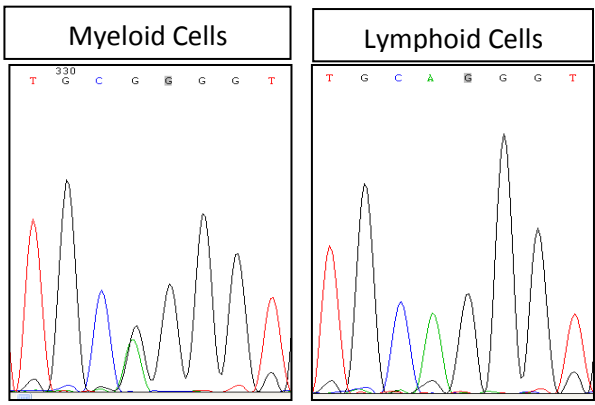

B)

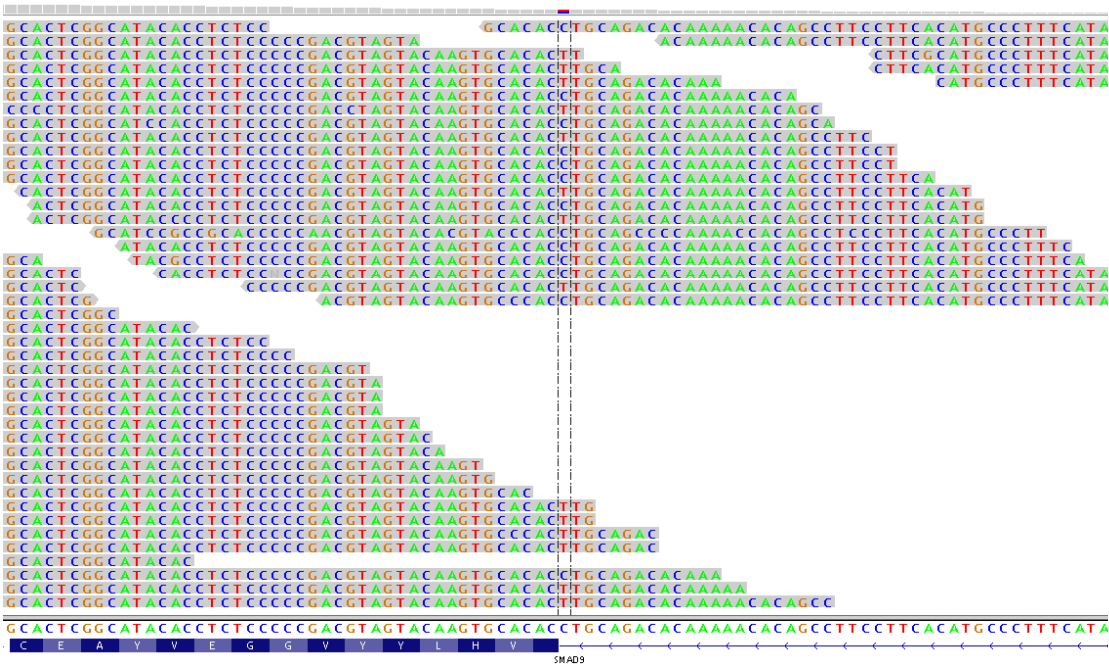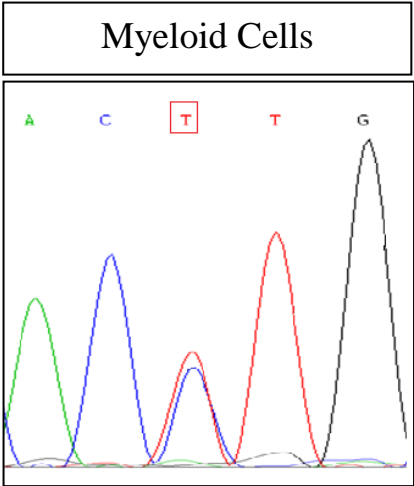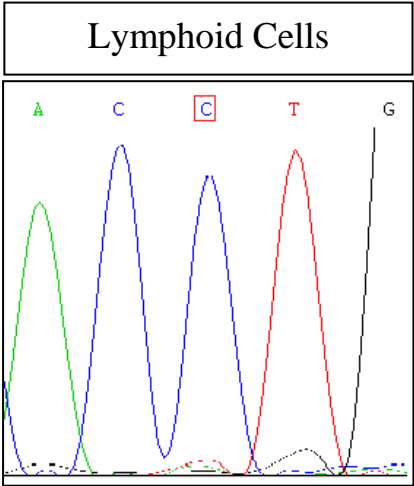

C)

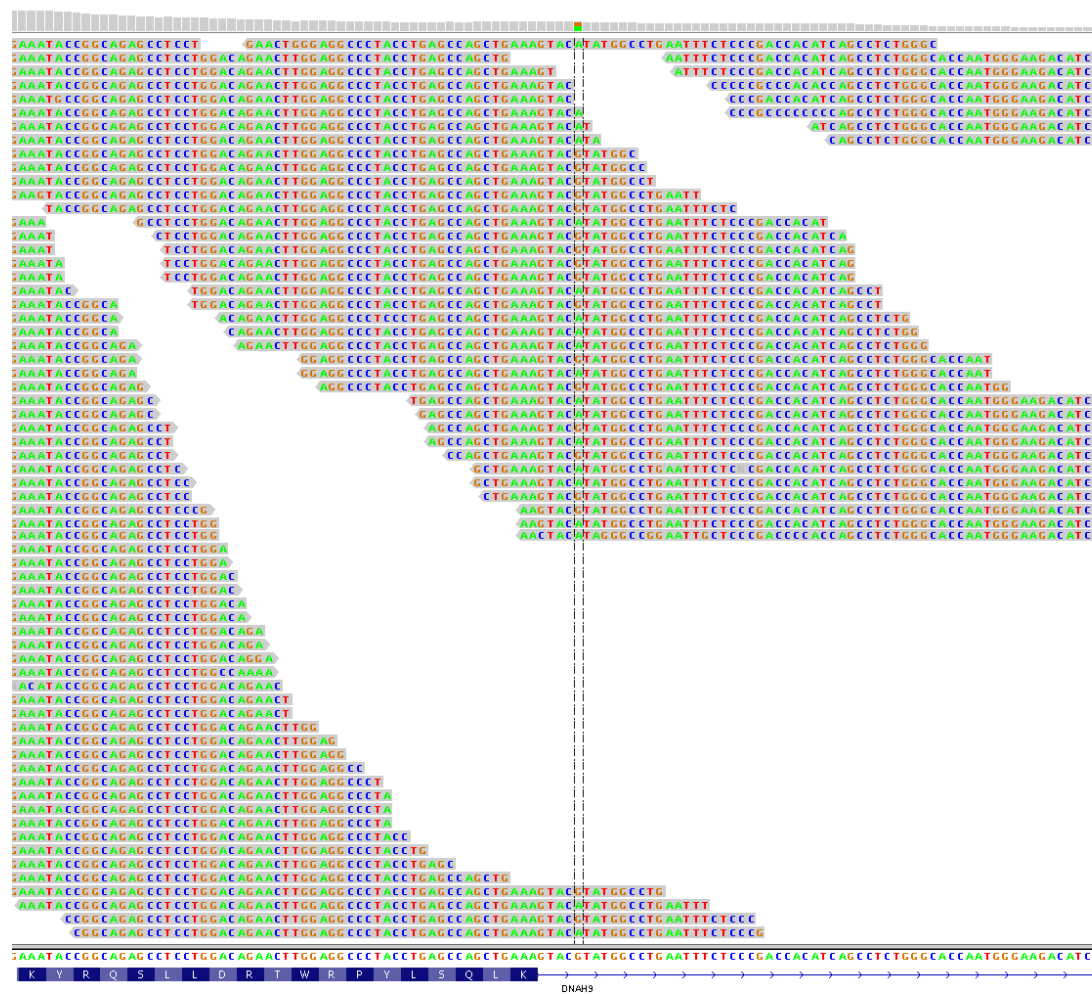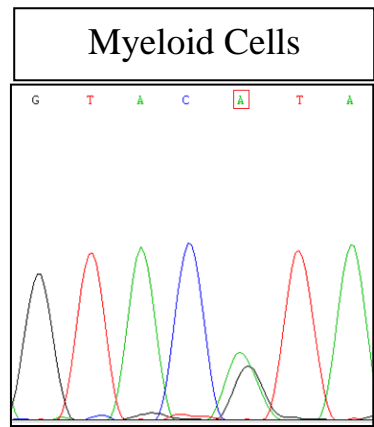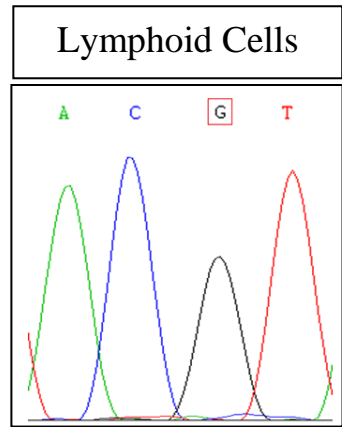

Supplement: Supplementary file 4 [file mgg30001-0246-SD4.pdf]

Suppl Fig. 5

A)

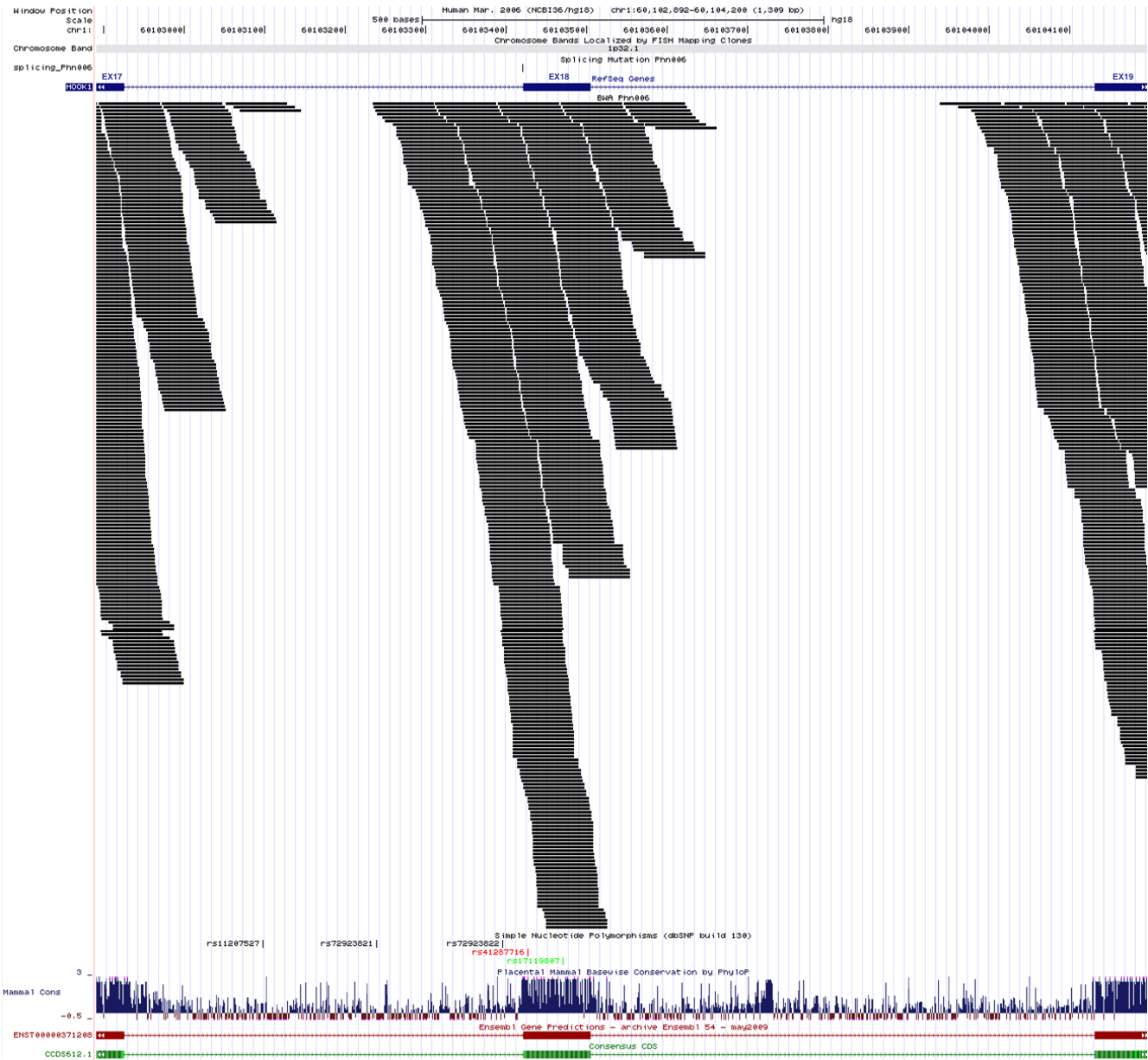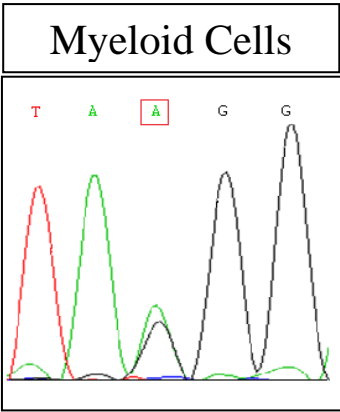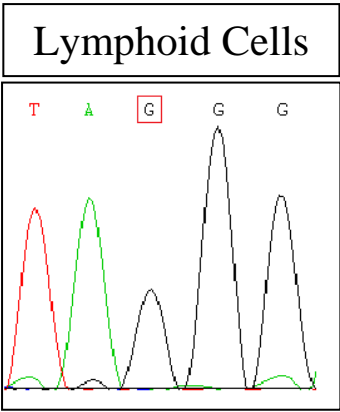

B)

Ph-001

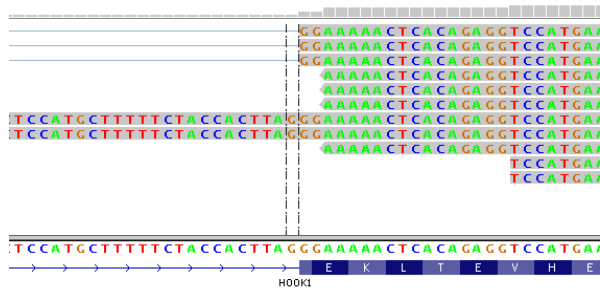

Ph-002

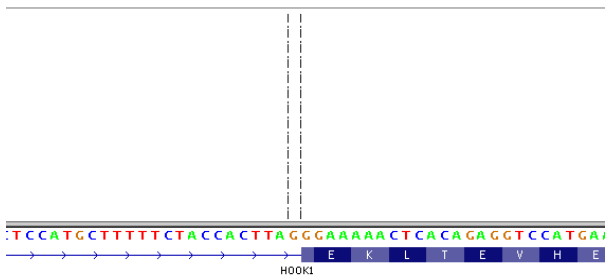

Ph-003

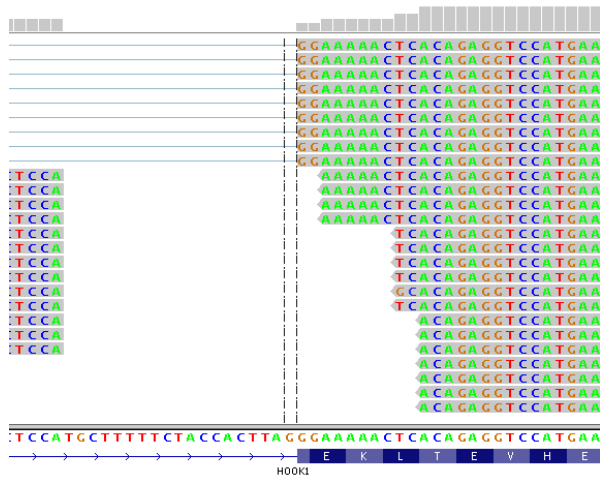

Ph-004

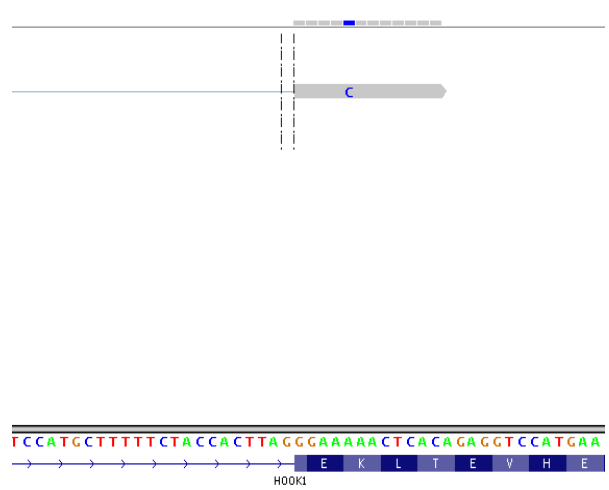

Ph-005

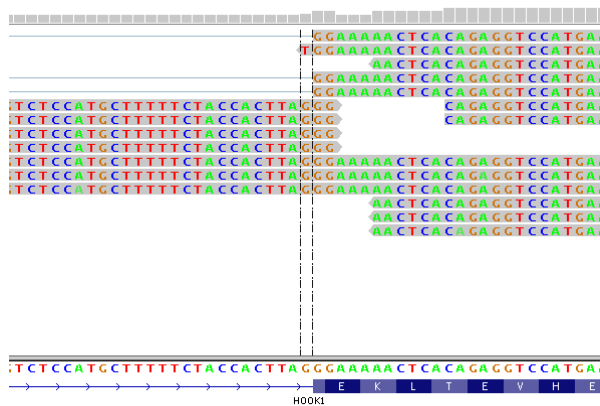

Ph-007

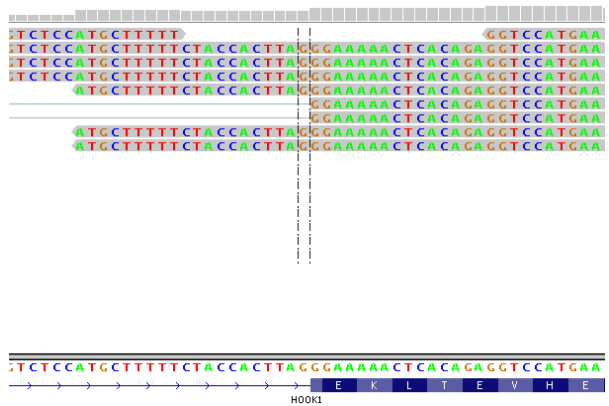

Ph-008

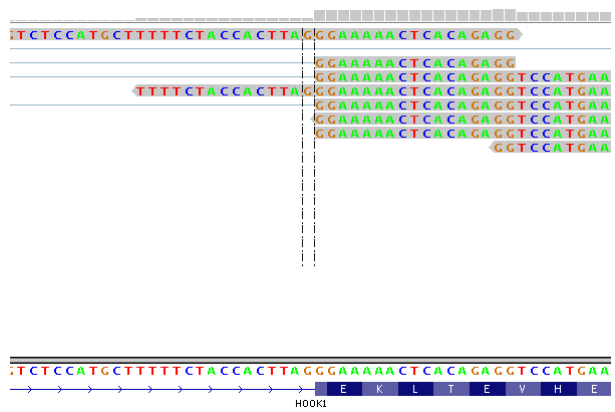

Supplement: Supplementary file 5 [file mgg30001-0246-SD5.pdf]

Suppl Fig. 6

A)

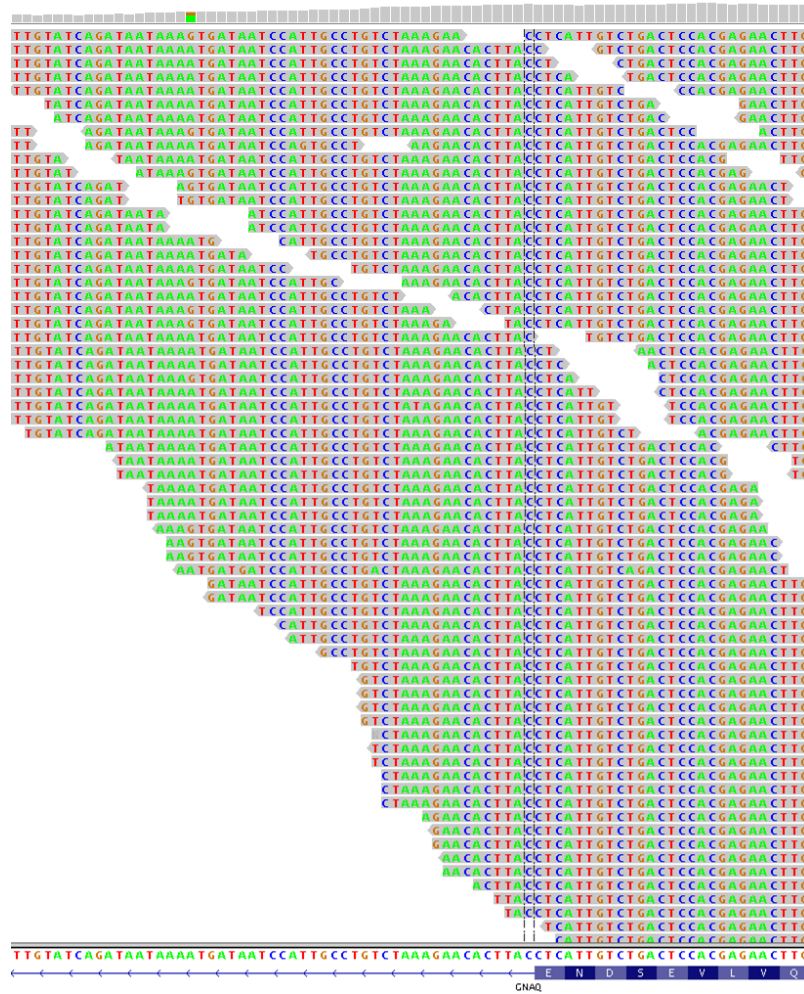

B)

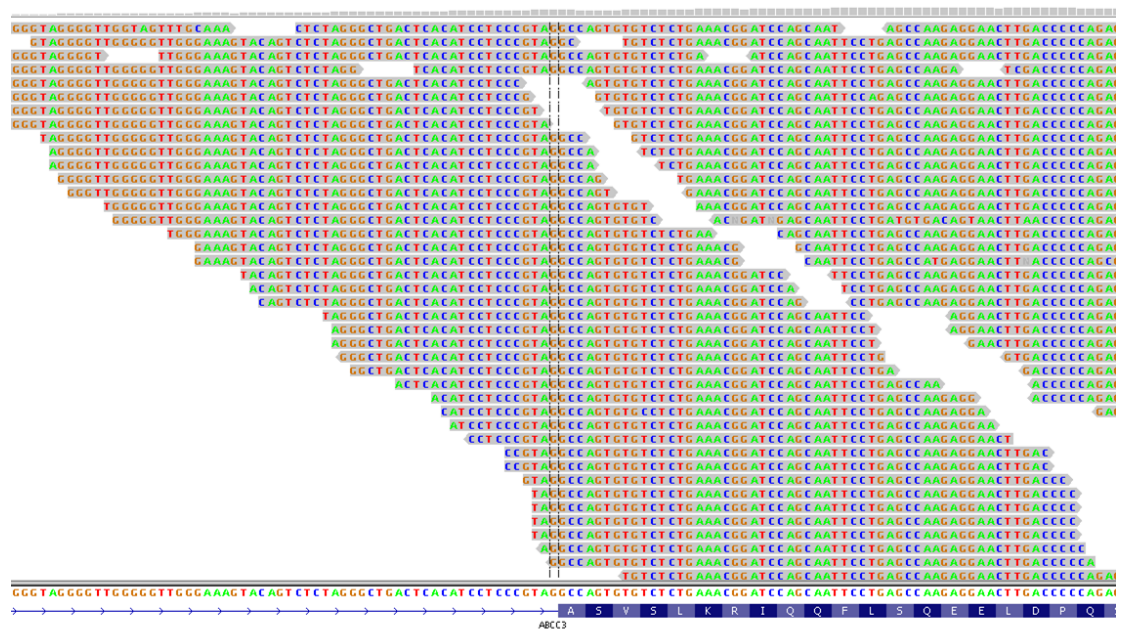

C)

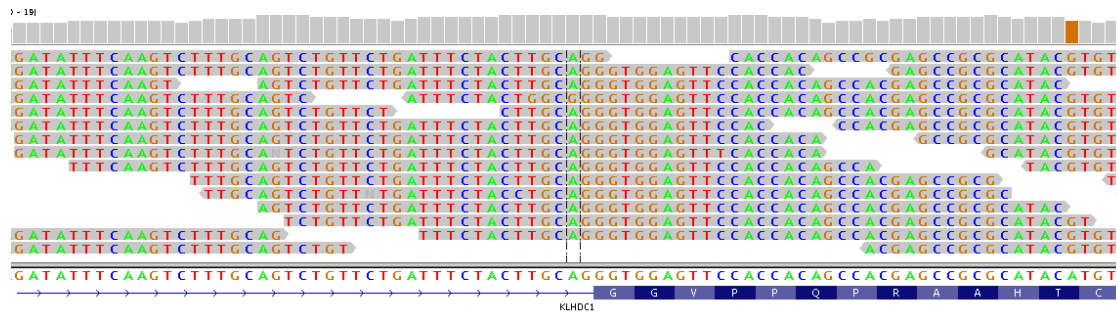

D)

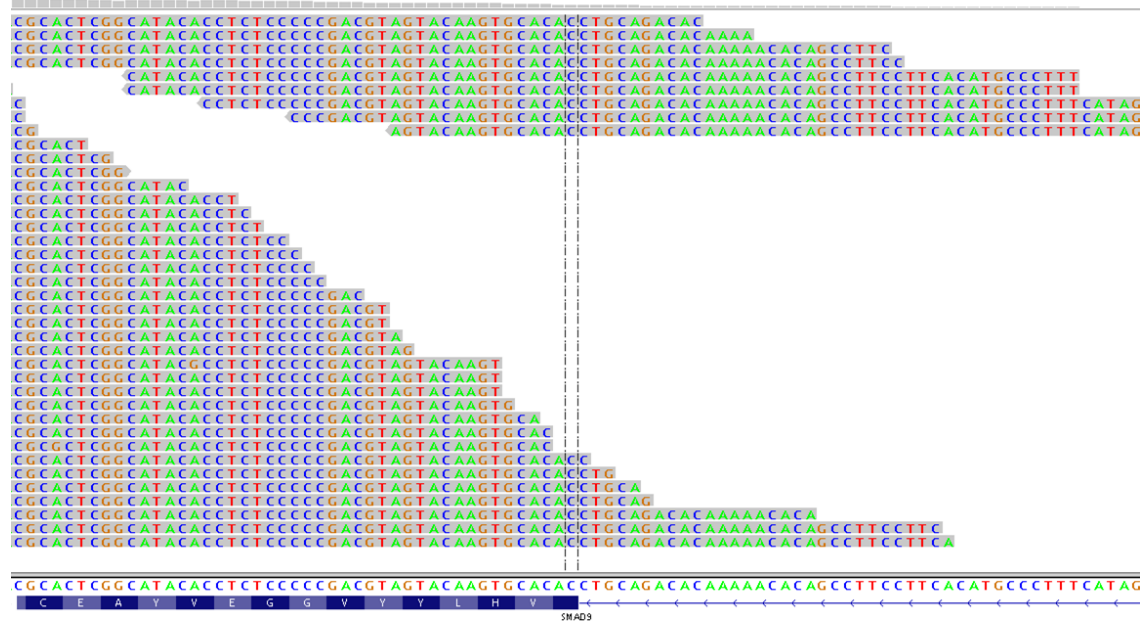

E)

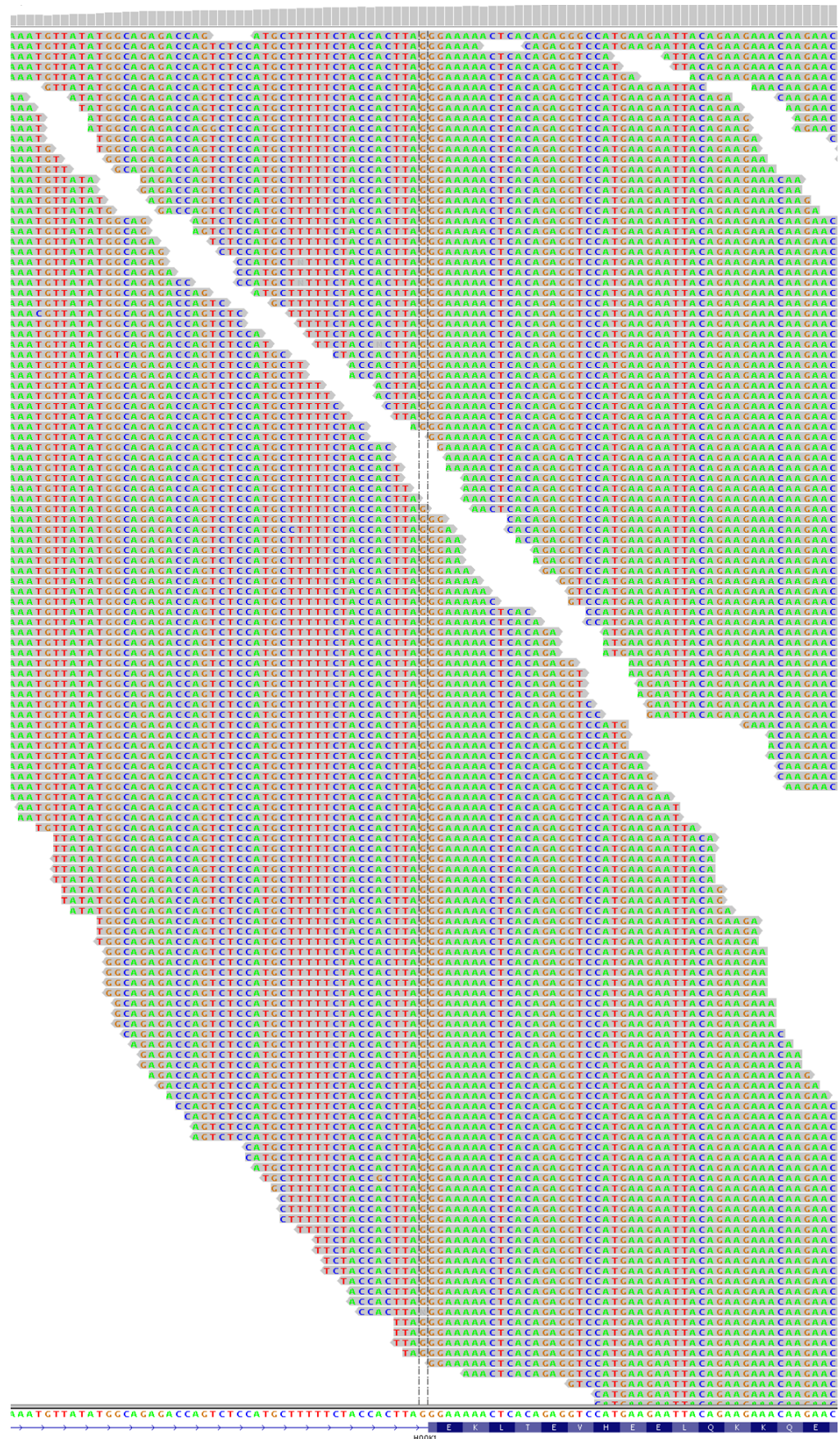

F)

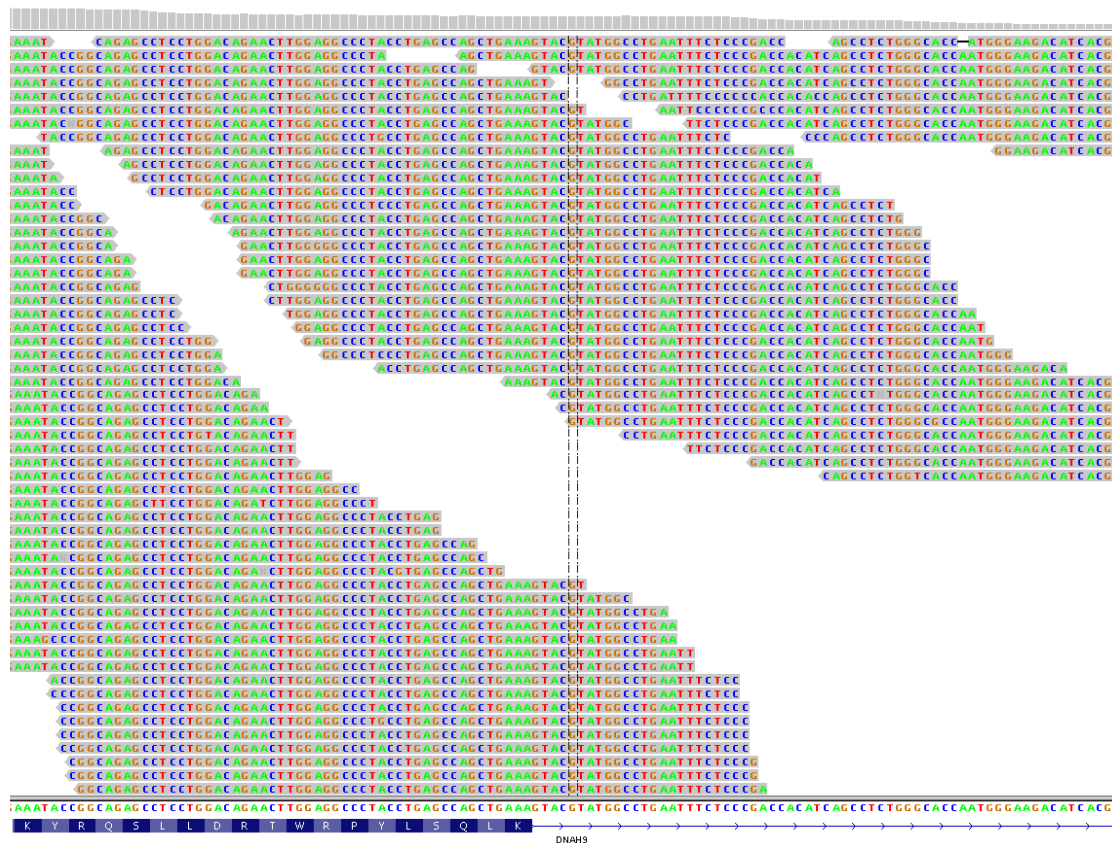

G)

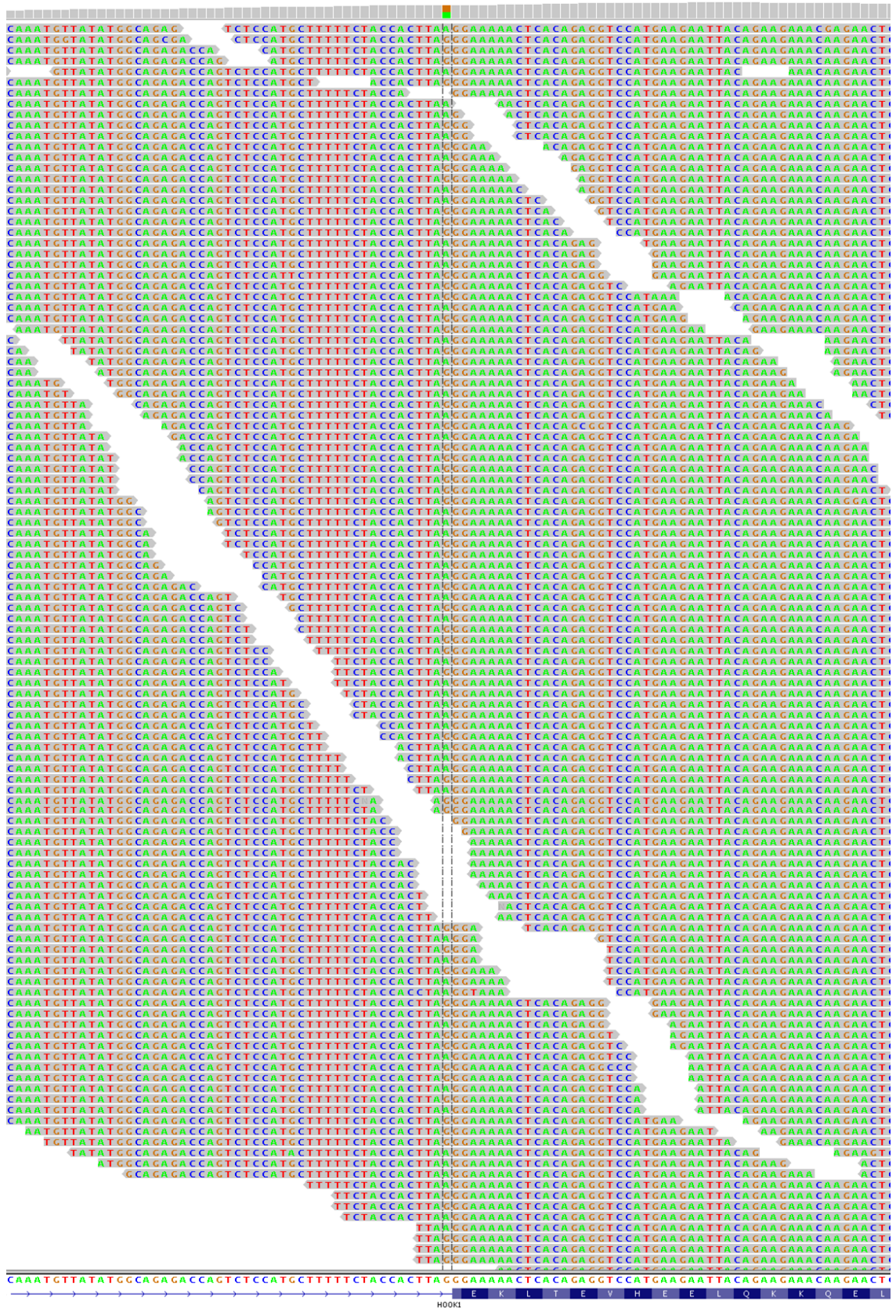

Supplement: Supplementary file 6 [file mgg30001-0246-SD6.pdf]
