# Supplementary material for: Identification of novel point mutations in splicing sites integrating whole-exome and RNA-seq data in myeloproliferative diseases
Source: Mol Genet Genomic Med. 2013 Jul 7;1(4):246–59. doi: 10.1002/mgg3.23 (PMC3865592; doi:10.1002/mgg3.23)
Supplement: Supplementary file 13 [file mgg30001-0246-SD13.doc]

Suppl. Tab. 1

a)

| Annovar annotation | |  |  |  |  |  |  |
| --- | --- | --- | --- | --- | --- | --- | --- |
| ID | Genic regions | | | | Non-genic regions | | TOT |
|  | Exons | Splicing | UTRs | Introns | Intergenic | ncRNA |  |
| Ph+001 | 4 | 1 | 0 | 0 | 4 | 3 | 12 |
| Ph+002 | 7 | 2 | 1 | 12 | 2 | 0 | 24 |
| Ph+003 | 236 | 96 | 242 | 893 | 404 | 93 | 1964 |
| Ph+004 | 127 | 48 | 171 | 408 | 214 | 55 | 1023 |
| Ph+005 | 146 | 64 | 131 | 239 | 156 | 41 | 777 |
| Ph+006 | 546 | 110 | 174 | 499 | 315 | 105 | 1749 |
| Ph+007 | 64 | 32 | 80 | 135 | 112 | 23 | 446 |
| Ph+008 | 11 | 0 | 1 | 9 | 6 | 0 | 27 |

b)

| Annovar annotation | |  |  |  |  |  |  |
| --- | --- | --- | --- | --- | --- | --- | --- |
| ID | Genic regions | | | | Non-genic regions | | TOT |
|  | Exons | Splicing | UTRs | Introns | Intergenic | ncRNA |  |
| Ph-001 | 13 | 2 | 1 | 4 | 0 | 0 | 20 |
| Ph-002 | 33 | 9 | 6 | 26 | 73 | 21 | 168 |
| Ph-003 | 11 | 0 | 0 | 1 | 1 | 1 | 14 |
| Ph-004 | 11 | 1 | 10 | 33 | 26 | 4 | 85 |
| Ph-005 | 22 | 1 | 8 | 17 | 31 | 2 | 81 |
| Ph-006 | 14 | 4 | 10 | 47 | 51 | 16 | 142 |
| Ph-007 | 11 | 1 | 8 | 5 | 12 | 1 | 38 |
| Ph-008 | 12 | 2 | 11 | 9 | 19 | 4 | 57 |
