# Supplementary material for: Identification of novel point mutations in splicing sites integrating whole-exome and RNA-seq data in myeloproliferative diseases
Source: Mol Genet Genomic Med. 2013 Jul 7;1(4):246–59. doi: 10.1002/mgg3.23 (PMC3865592; doi:10.1002/mgg3.23)
Supplement: Supplementary file 14 [file mgg30001-0246-SD14.doc]

Suppl. Tab. 2.

| **GENE** | **FORWARD PRIMERS (5'--3')** | **REVERSE PRIMERS (5'--3')** |
| --- | --- | --- |
| ABCC3 | GAGTCACAGGTGGTCAGGTCGGGTG | CGGAAGAGGGAGGAGGTCAGAGTCG |
| KLHDC1 | GTG TGT GTG AAA GAG AAA CAG AGG AG | TCG TGG CTG TGG TGG AAC TC |
| HOOK1 | CTA AAG ATT CAG CCT CCC TAC TA | TCA TTT TAT TAG ATT TTT TGC TTT A |
|  | GCC TGA CTG TTT ACA TCC TCA T | ATA CTG TTG TCT GAT CCA AGT TT |
| SMAD9 | CAG GGT GCT TTT TTG TTT TG | GTA GAG AAG GGG TTT CAC TGT G |
|  | GAA GCC GTG TTG ATA GTT GC | TTT AGG TCA CAC ATC TCA TTC ATC |
| GNAQ | AAT GAA AAC AAA ACA AAA CAG GA | GTG TTA CCC AGA ATG TTT TAA CTT |
|  | CAC AAA GAC CTG CTC ACA CA | TTT CCC TAA GTT TGT AAG TAG TGC |
| DNAH9 | CCA AAG TGC TGG AAT TAC AGG | GTC TAA GCC CTT TTG CCT CAG |
|  | GCT CTC AAT CTC AAG AAG CG | CAG AGC AGA GTC TCA CCT GAT G |
