# Supplementary material for: Identification of novel point mutations in splicing sites integrating whole-exome and RNA-seq data in myeloproliferative diseases
Source: Mol Genet Genomic Med. 2013 Jul 7;1(4):246–59. doi: 10.1002/mgg3.23 (PMC3865592; doi:10.1002/mgg3.23)
Supplement: Supplementary file 15 [file mgg30001-0246-SD15.doc]

| **Symbol** | **Gene name** | **Description** | **Gene Ranker**  **Score** | **Cancer** |
| --- | --- | --- | --- | --- |
| GNAQ | Guanine Nucleotide Binding Protein (G Protein), Q Polypeptide | Regulates B-cell selection and survival and is required to prevent B-cell-dependent autoimmunity. Regulates chemotaxis of BM-derived neutrophils and dendritic cells. | **1.5** | Endometrium; Eye; Kidney; Large Intestine; Lung; Ovary; Prostate; Skin |
| ABCC3 | ATP-Binding Cassette, Sub-Family C (CFTR/MRP), Member 3 | Member of the superfamily of ATP-binding cassette (ABC) transporters. ABC transport various molecules across extra- and intra-cellular membranes. Member of the MRP subfamily which is involved in multi-drug resistance. | **4.75** | Breast; Central Nervous System; Endometrium; Kidney; Large Intestine; Liver; Lung; Prostate; Skin; Upper aerodigestive tract; Urinary Tract |
| KLHDC1 | Kelch Domain-Containing Protein 1 | Transcribed at high levels in skeletal muscle and at low levels in most other tissues tested; expression was absent in peripheral blood leukocytes. KLHDC1 is primarily localized to the cytoplasm. | - | Kidney; Large Intestine; Lung; Ovary; Pancreas |
| SMAD9 | SMAD Family Member 9 | Member of the SMAD family, which transduces signals from TGF-beta family members. | **0.5** | Cervix; Kidney; Large Intestine; Lung |
| HOOK1 | Hook Homolog 1 (Drosophila) | Member of the hook family of coiled-coil proteins, which bind to microtubules and organelles through their N- and C-terminal domains, respectively. The encoded protein interacts with several members of the Rab GTPase family involved in endocytosis. | **0.5** | Biliary tract; Breast; Endometrium; Kidney; Large Intestine; Lung; Ovary; Urinary tract |
| DNAH9 | Dynein, Axonemal, Heavy Chain 9 | This gene encodes the heavy chain subunit of axonemal dynein, a large multi-subunit molecular motor. | **1** | Autonomic ganglia; Breast;Central nervous system; Endometrium; Haematopoietic and lymphoid tissue*; Kidney; Large intestine; Lung; Ovary; Pancreas; Prostate; Skin; Upper aerodigestive tract; Urinary tract |

Suppl. Tab 3
